# Collective cooperative intelligence

**DOI:** 10.1073/pnas.2319948121

**Published:** 2025-06-16

**Authors:** Wolfram Barfuss, Jessica Flack, Chaitanya S. Gokhale, Lewis Hammond, Christian Hilbe, Edward Hughes, Joel Z. Leibo, Tom Lenaerts, Naomi Leonard, Simon Levin, Udari Madhushani Sehwag, Alex McAvoy, Janusz M. Meylahn, Fernando P. Santos

**Affiliations:** ^a^Argelander-Chair Integrated System Modeling for Sustainability Transitions, Center for Development Research (ZEF), University of Bonn, Bonn 53113, Germany; ^b^Santa Fe Institute, Santa Fe, NM 87501; ^c^Max-Planck-Institute for Evolutionary Biology, 24306 Plön, Germany; ^d^Center for Computational and Theoretical Biology, Julius-Maximilians University, Würzburg 97074, Germany; ^e^Department of Computer Science, University of Oxford, OX1 3QD Oxford, United Kingdom; ^f^Google DeepMind, N1C 4DN London, United Kingdom; ^g^Machine Learning Group, Département d’Informatique, Université Libre de Bruxelles, 1050 Bruxelles, Belgium; ^h^Artificial Intelligence Lab, Computer Science department, Vrije Universiteit Brussel, 1050 Brussels, Belgium; ^i^Center for Human Compatible AI, University of California, Berkeley, CA 94720; ^j^Department of Mechanical and Aerospace Engineering, Princeton University, Princeton, NJ 08544; ^k^Department of Ecology and Evolutionary Biology, Princeton University, Princeton, NJ 08544; ^l^Department of Computer Science, Stanford University, Stanford, CA 94305; ^m^School of Data Science and Society, University of North Carolina at Chapel Hill, Chapel Hill, NC 27599; ^n^Department of Mathematics, University of North Carolina at Chapel Hill, Chapel Hill, NC 27599; ^o^Department of Applied Mathematics, University of Twente, 7522 NB Enschede, The Netherlands; ^p^Faculty of Science, Informatics Institute, University of Amsterdam, 1098 XH Amsterdam, The Netherlands

**Keywords:** cooperation, collective action, complex systems science, multiagent reinforcement learning

## Abstract

Cooperation at scale is critical for achieving a sustainable future for humanity. However, achieving collective, cooperative behavior—in which intelligent actors in complex environments jointly improve their well-being—remains poorly understood. Complex systems science (CSS) provides a rich understanding of collective phenomena, the evolution of cooperation, and the institutions that can sustain both. Yet, much of the theory in this area fails to fully consider individual-level complexity and environmental context—largely for the sake of tractability and because it has not been clear how to do so rigorously. These elements are well captured in multiagent reinforcement learning (MARL), which has recently put focus on cooperative (artificial) intelligence. However, typical MARL simulations can be computationally expensive and challenging to interpret. In this perspective, we propose that bridging CSS and MARL affords new directions forward. Both fields can complement each other in their goals, methods, and scope. MARL offers CSS concrete ways to formalize cognitive processes in dynamic environments. CSS offers MARL improved qualitative insight into emergent collective phenomena. We see this approach as providing the necessary foundations for a proper science of collective, cooperative intelligence. We highlight work that is already heading in this direction and discuss concrete steps for future research.

Cooperation is the ability of a group to successfully and voluntarily act together toward a common interest, even when short-term or individual gains make selfish behavior more appealing to individuals (1). Such situations are commonly referred to as social dilemmas. In a social dilemma, every actor has the incentive to behave selfishly, yet everyone would be better off if all behaved cooperatively. Cooperation is required to maintain environmental commons, local and global, e.g., fisheries, the atmosphere, and biodiversity, but also social commons, such as public infrastructure, education, and healthcare.

The study of whether, when, and how cooperation emerges is an interdisciplinary pursuit involving research fields as diverse as biology, physics, computer science, engineering, and different branches of social sciences. Established mechanisms to achieve and maintain cooperation include outside authorities (2) and bottom–up arrangements based on social reciprocity (3). External authorities can resolve social dilemmas by installing a punishment or reward scheme, e.g., via taxes and subsidies, that makes selfish actions less attractive to individuals, whereas bottom–up arrangements and social reciprocity find a way to punish defecting behavior through peers (4). However, the challenge of cooperation is far from being solved.

First, large collectives complicate the emergence and robustness of cooperation. Although many mechanisms have been identified that support its emergence and maintenance, it is also widely recognized that effective scaling mechanisms are rare (5): in global public goods, such as the climate, there is no single outside actor with sufficient enforcement power to ensure cooperation authoritatively. In situations involving many, mostly anonymous, participants, reciprocity mechanisms are hard to stabilize (6). Hence, a key challenge for future research remains identifying principles of collective information processing and collective action that offer robust pathways to cooperation in large groups and across scales of organization.

Second, the complexity and variety of human behavior complicate the challenge of cooperation. A more comprehensive understanding of human behavior is required because of its importance in shaping future pathways to sustainability (7). Securing a livable planet and the well-being of future generations requires changes in policy, technology, and, not least, individual behavior. Human decision-making must be conceptualized as a multifaceted cognitive process coevolving with local and global contexts (8). Furthermore, situations involving diverse human actors with heterogeneous needs, preferences, and characteristics are critical to sustainable futures and present a significant challenge to cooperation (9). It is unknown how the diversity among actors and the cognitive complexity within them can offer robust principles for cooperation.

Third, the complexity of the environments in which the actors are embedded makes stabilizing cooperation challenging (10). Challenges arise from 1) feedback (both gradual and abrupt) between the environment and actors’ choices; 2) delayed or sparse consequences of actions; and 3) multiple kinds of risks and uncertainties in the environment (11). It is unresolved which factors of dynamic environmental contexts offer robust pathways toward cooperation.

Fourth and last, transient dynamics complicate the challenge of cooperation. While many cooperation-promoting mechanisms have been found, little attention has been paid to transient phenomena toward cooperation (12). This includes time scales on which cooperation is achievable, the critical transition points at which cooperation becomes viable, and the stability and resilience of a cooperative arrangement. All these elements are crucial to consider for sustainability transitions.

To address these challenges, mathematical models are essential. Process-based, mechanistic models allow for theory building and in silico experimentation when experimental approaches are either too costly or infeasible (13). The complex systems science (CSS) approach has produced a robust understanding of how interactions among simple identical agents can create macrolevel structures and properties. Notable fields include swarm intelligence, evolutionary games, and population dynamics. However, the microscale individual level in biological, social, and artificial systems consists of sophisticated entities with behaviors and interactions that may not be well-characterized by simplistic representations (14). It is an open question of what collective behavior emerges when interacting entities are capable of sophisticated cognition and embedded in an environmental context.

Agent-based modeling and the field of artificial life allow for complex individual decision rules and agent heterogeneity (15). Yet, this additional complexity often means that the models cannot be analyzed analytically and must be simulated. In addition, many agent-based models suffer from the well-documented criticism of “garbage-in-garbage-out” because the models are easily overparameterized, and the rules afforded to the agents are not necessarily principled nor empirically grounded (16). Multiagent reinforcement learning (MARL) can be viewed as a type of agent-based modeling in which agent behavior does not have to be fixed in advance with plausible heuristics (17). Instead, the agents themselves learn how to behave. However, MARL simulations are usually highly stochastic and computationally expensive, and the often large number of free parameters can make them challenging to interpret (18).

In this paper, we argue that bridging the communities of CSS and MARL can help with these challenges. MARL operationalizes complex individual cognition in dynamic environments. Conversely, CSS enables a principled understanding of the emergence of cooperation, bringing rigorous ideas about emergence and dynamics to MARL for improved interpretation. This is how we understand Collective Cooperative Intelligence. Intelligence refers to the general ability (mainly of a single actor) to achieve a diverse set of goals (19), which is formalized in AI research. Collective intelligence is the intelligence of a group of primarily simple individuals, as primarily studied in CSS (20). Cooperative intelligence is the ability to achieve cooperation in a wide range of contexts (21). Thus, Collective Cooperative Intelligence is the ability of a collective—composed of individuals capable of intelligent decision-making—to act together to improve their common welfare by solving or identifying problems posed by the environment. With AI rapidly advancing, it is imperative to bring these perspectives together (22). Creating tractable models of collective reinforcement learning is crucial for a better understanding of the fundamental principles that drive the emergence of cooperation among complex agents in dynamic environments. Such an understanding can help researchers design more cooperative algorithms and environments and identify critical leverage points toward a sustainable future.

## Background

Here, we juxtapose CSS and MARL approaches to the challenge of cooperation. Their relative differences are summarized in Table 1 while *SI Appendix* provides more details.

**Table 1.** Comparison between CSS and MARL approaches to the study of cooperation problems

	CSS	MARL
Goal	First understand cooperation, then improve it	First improve cooperation, then understand it
Scope	1) Low-dimensional environment	1) High-dimensional environment
	2) Cooperative behavior available	2) Cooperative behavior to be learned
Typical	1) Level of cooperation	1) Total social welfare
performance	2) Mechanism plausibility	2) Algorithm scalability
criteria	3) Mechanism simplicity	3) Generalization
Methodological	1) Dynamics	1) Algorithm design
tools	2) Analytics	2) Simulations
Comparative	1) Analytically explainable	1) Agent heterogeneity
advantages	2) Computationally lightweight	2) Applicable to large-scale environments

### CSS.

Complex systems are generally out of equilibrium with many interacting components, feedback, and couplings between components and levels (23). CSS has been instrumental in studying cooperation through formal models (24). The primary goal of CSS is to understand how macrolevel cooperation emerges from and interacts with simple microlevel processes, before intervening to improve cooperation.

Typically, models aim to explain emerging cooperation from simple yet plausible mechanisms where “cooperation” is based on an atomic action with the property that more cooperation translates to higher social welfare (25). For example, the famous strategy tit-for-tat, which merely reciprocates what the opponent did in the previous turn, is surprisingly successful against much more complicated strategies (26). CSS methods are diverse. Typical methods used are evolutionary game theory (27), nonlinear dynamics (28), and complex social network models (29). With its academic origins in theoretical physics and mathematical biology (30), the CSS approach to study cooperation is rooted in dynamics and often captures the analytic conditions for cooperation to emerge (31), which provides a thorough and robust understanding of the cooperation-promoting mechanisms at play. This is facilitated by making the models as simple as possible, which yields low-dimensional models compared to typical MARL simulations.

However, much of the existing theory in this field neglects the complexity of individuals and environmental contexts (14, 32). Humans are capable of foresight, have a theory of mind, make inferences about their environment, and can adapt their behavior correspondingly. There is a clear interest in adapting CSS models to accommodate these nuances (33–36). Considering how such models could be informed by MARL has great potential to unleash novel ways of modeling complex systems to tackle the challenges of collective cooperation in more complex settings.

### MARL.

In a typical MARL setting, each agent observes (parts of) the current state of the environment, then takes an action, after which they observe (part of) the new state of the environment and are provided with a reward indicating how desirable the previous “state–action–state” transition was. Over time, the agents update their strategies (a mapping from observation histories to probability distributions over their action space) to optimize the long-term amount of reward they receive (37). Studies of cooperation in MARL fall under the umbrella of Cooperative AI (21) with the overarching aim to improve the cooperative capabilities of advanced AI systems by prescribing how agents should (learn to) act. Extending machine learning interpretability techniques to MARL is an ongoing effort to advance also the understanding of MARL systems (38).

In contrast to CSS studies, cooperation is typically not readily available as an action. Instead, what constitutes a cooperative strategy, and how such a behavior can be implemented, must be learned from scratch (39) based on the performance criterion of total social welfare. Furthermore, the learning algorithms developed in MARL should generalize to novel situations and scale to high-dimensional environments. Modern MARL is inspired by a number of fields, including neuroscience, psychology, economics, and machine learning (40–43). For example, the commonly used idea of temporal-difference learning is based upon reward-prediction errors, common to humans, other animals, and machines. In recent years, these traditional ideas have been combined with advances in deep learning, achieving remarkable successes (18). Thus, the methodological focus often lies in designing novel algorithmic features to improve the cooperativeness of RL algorithms in large-scale computer simulations.

However, MARL simulations—on their own—do not facilitate analytically reliable insights into how collective cooperation emerges from complex human and machine behavior in dynamic environments. They often require significant computational resources, while the space to explore suffers from the curse of dimensionality. Moreover, they are typically highly stochastic in nature, and results can be difficult to interpret (18). We believe that a unified approach that combines approaches from CSS and MARL could fill this gap.

## Building Bridges

Here, we discuss how CSS and MARL can complement each other. First, we establish a shared language by treating MARL as a complex dynamical system. Second, we illustrate how CSS can offer MARL improved qualitative insight into emergent collective learning dynamics via its mathematical toolbox. Third, we discuss the potential of MARL for improving CSS approaches regarding formalizing collective behavior from cognitive processes in dynamic environments. We highlight steps for future research throughout the discussion, which we summarize in Box 3.

### Collective Reinforcement Learning Dynamics (CRLD).

Using nonlinear dynamical systems to model MARL is an interdisciplinary pursuit involving perspectives from economics (42, 45), sociology (46, 47) machine learning (48, 49), control theory and engineering (50, 51), statistical mechanics (52, 53), and mathematical biology (54, 55). These fields have diverse research goals. Economics focuses on convergence to equilibrium solutions, whereas statistical mechanics is interested in nonconvergence scenarios. Machine learning and engineering care for the development of scalable algorithms and evaluation metrics. Mathematical biology and sociology view reinforcement learning as a model of biologically plausible adaptation and study when and how certain learning processes serve as a model of human behavior. To overcome disciplinary divides, we summarize the intersection of these approaches under the term CRLD and present an exemplary illustration in Box 1.

Box 1. Collective Reinforcement Learning Dynamics.

CRLD provides a bridge between CSS and MARL based on the mathematical framework of MARL:

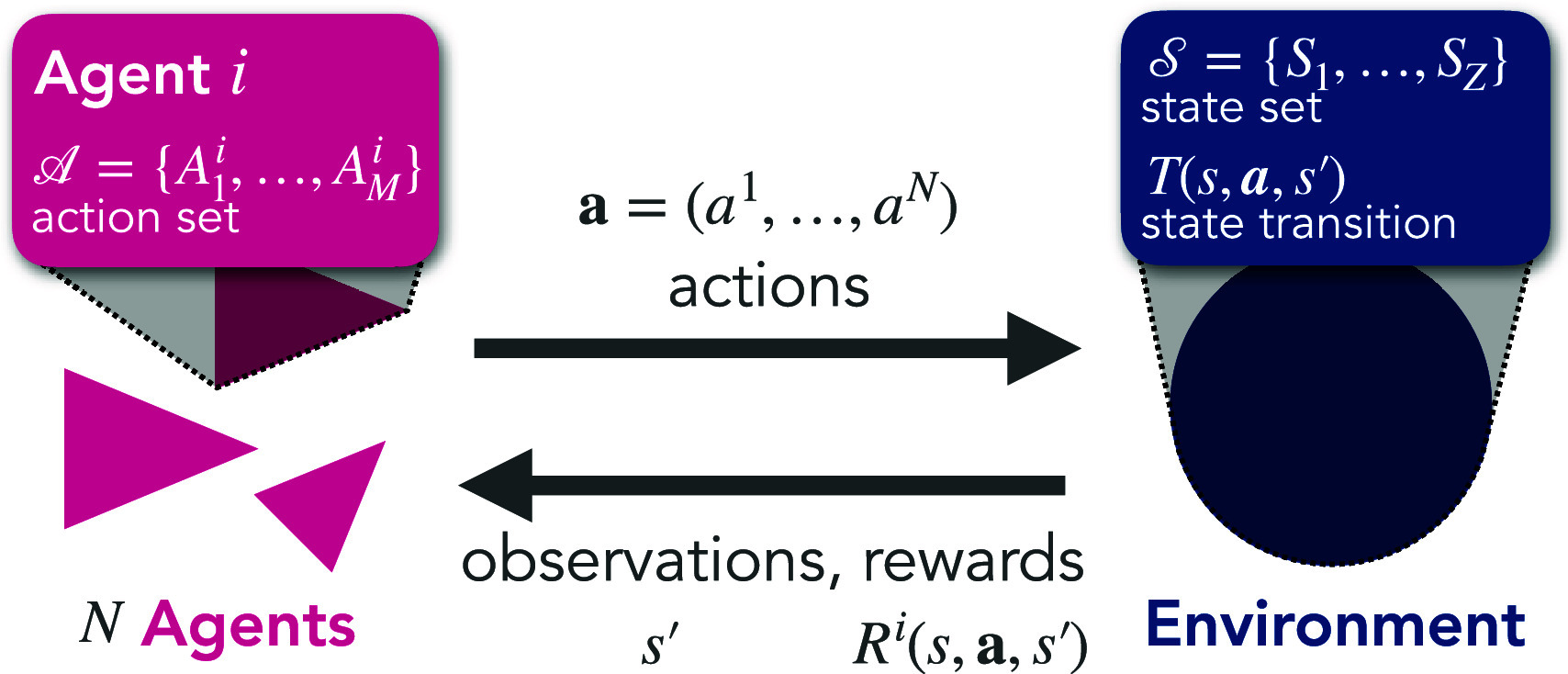

At each point in time, each agent i∈ {1, ..., N} can choose an action from its action set $A^{i}$. These agents are embedded in a (physical, ecological, or social) environment with possible states S. States change according to the environmental transition function: a probability of entering the next state $s^{\prime }$, for each state *s* and each combination of agents’ actions **a**. Agents receive external rewards: a numeric value for each agent *i*, for each transition from *s* via action **a** to state $s^{\prime }$. Instead of RL algorithms, CRLD uses a set of dynamical equations, modeling the learning of the agents. As with algorithms, many variants exist. Here, we illustrate the dynamic equations of temporal-difference learning (44):

$$\begin{array}{c} X_{t+1}^{i}\left(s,a\right)=\frac{1}{Z_{X_{t}}^{i}\left(s\right)}X_{t}^{i}\left(s,a\right)\exp(\eta^{i}\cdot \delta_{X_{t}}^{i}\left(s,a\right)) \end{array}$$

The joint strategy, i.e., the probability of each agent *i* choosing action *a* in state *s*, $X_{t+1}^{i}\left(s,a\right)$, is updated by the product of the previous strategy, $X_{t}^{i}\left(s,a\right)$, and the exponent of the previous strategy-average temporal-difference error, $\delta_{X_{t}}^{i}\left(s,a\right)$, multiplied with the effective learning rate $\eta^{i}$, and properly normalized, $1\;/\;Z_{X_{t}}^{i}\left(s\right)$, to yield a probability distribution. $\delta_{X_{t}}^{i}\left(s,a\right)$ tells the agents how to adapt their strategy to gain more reward over time, averaging out all randomness due to the stochasticity of agents’ strategies and environmental transitions. See *SI Appendix* for all details. The benefits of this framework are described below for MARL in Complex Phenomena and for CSS in Cognition in Contexts.

However, the study of cooperation has not been at the center of studies despite notable examples existing in mathematical biology and sociology (see *SI Appendix*). Here, we argue for putting the question of cooperation at the center of a research agenda on collective cooperative intelligence, accompanied by regarding learning as a biologically plausible model of human and AI behavior and focusing on the complex, transient learning dynamics. See Box 2 for a minimal application example.

Box 2. Example environment.

Here, we illustrate an environment that allows for the study of collective action under ecological tipping.

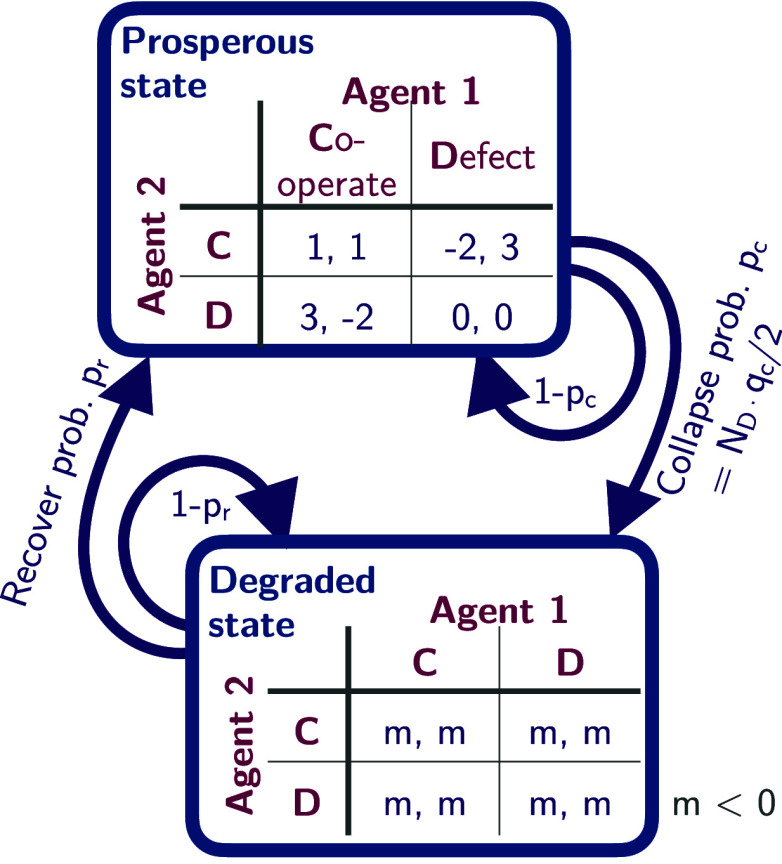

**Fig. 1.**
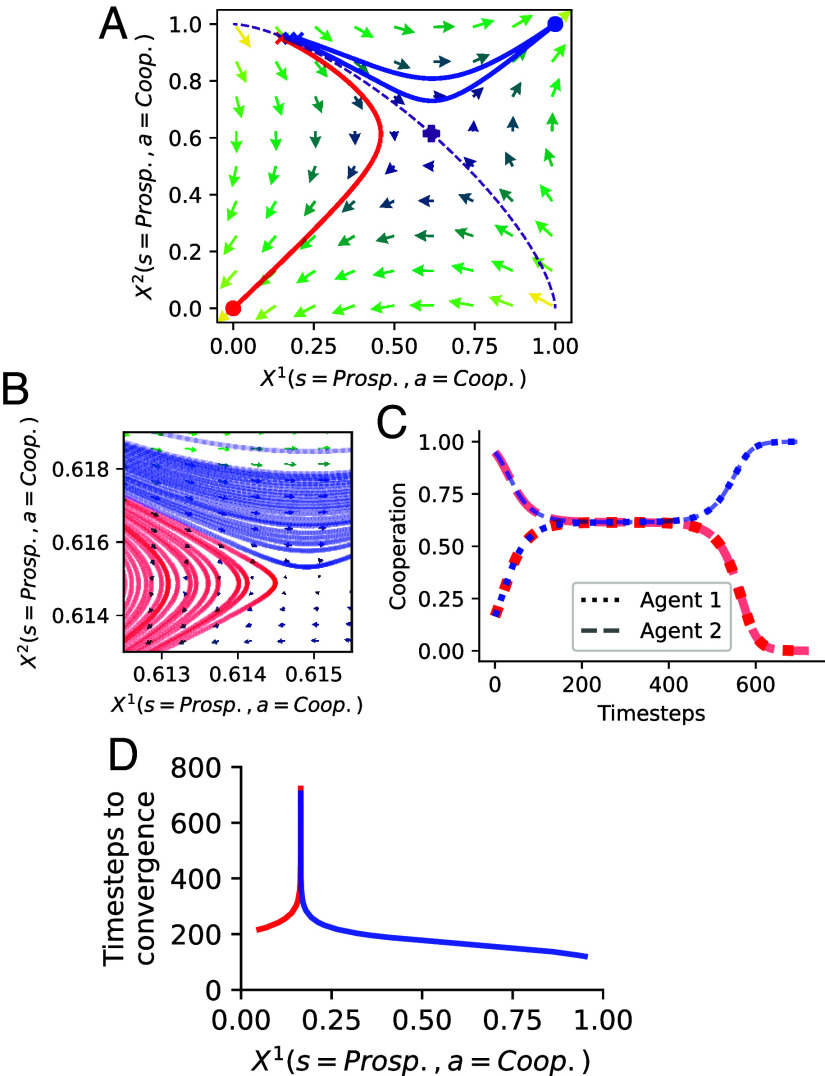
Multistability in CRLD (Box 1) applied to the ecological tipping environment (Box 2). (*A*) Phase space of the prosperous state. (*B*) Detailed bundle of learning trajectories. (*C*) Emergent timescales with abrupt transitions in transient dynamics. (*D*) Critical slowing down around the critical point. See *SI Appendix* for details.

**Fig. 2.**
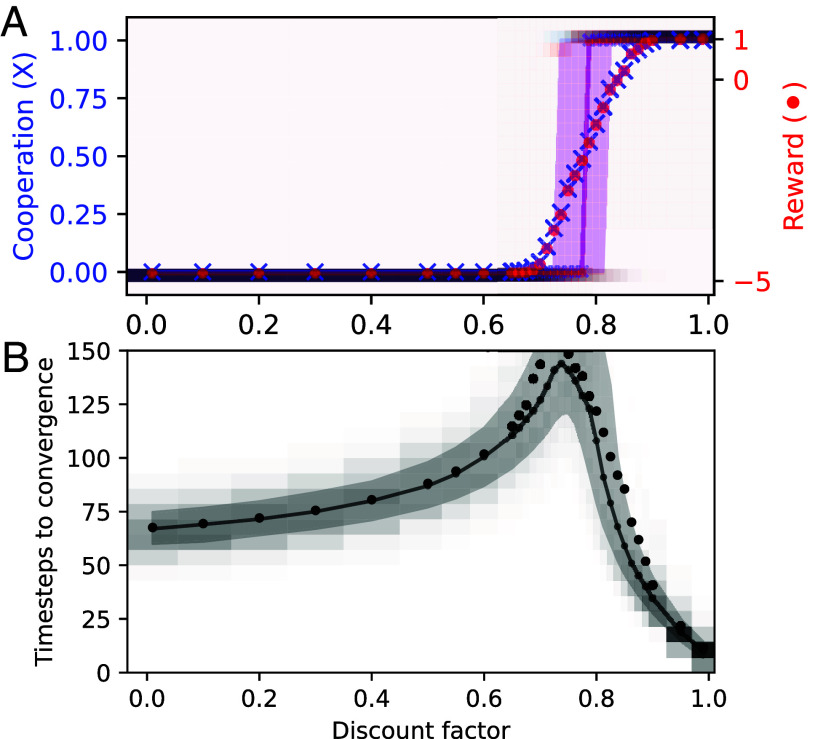
Critical transitions in CRLD (Box 1) applied to the ecological tipping environment (Box 2). (*A*) Cooperation levels and final rewards. (*B*) Time steps to convergence show a critical slowing down around the critical point. For each discount factor, both plots show a histogram of converged results from 250 random initial conditions by a color map, their mean by large markers, their median by small markers, and the range between lower and upper quartiles by the shaded regions. See *SI Appendix* for details.

**Fig. 3.**
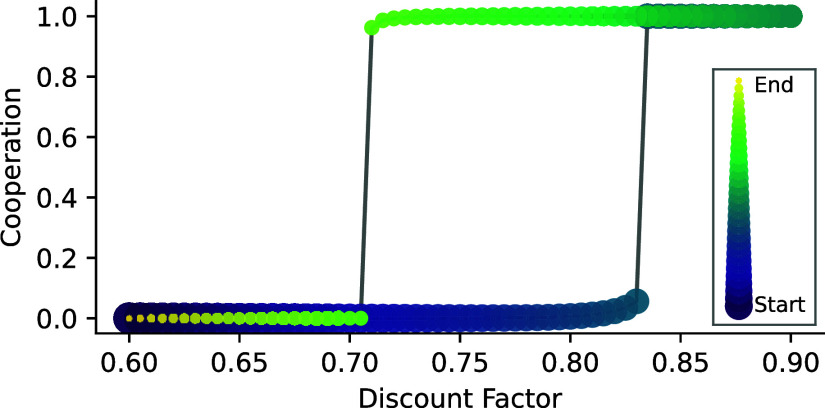
Hysteresis in CRLD (Box 1) applied to the ecological tipping environment (Box 2). The discount factor changes while the agents keep on learning. The size and color of the dots represent the time, from dark to light and from big to small as from past to future. See *SI Appendix* for details.

Two agents play a standard public goods game. Everyone is better off by cooperating but each agent has an immediate incentive to exploit the other. However, here, the agents are embedded in a dynamic environment consisting of two states, a prosperous and a degraded one. Each defecting agent increases the probability of collapse by an amount $q_{c}/2$. When collapsing to the degraded state, each agent suffers from the collapse by a negative impact *m* at each time point until they recover to the prosperous state, governed by the recovery probability $p_{r}$. For Figs. 1–3, we use $m=-5,q_{c}=0.2,q_{r}=0.01$.

Methodologically, CRLD studies are characterized by two forms of idealization. First, CRLD focuses on low-dimensional environments compared to typical MARL studies. Often, just static games with two agents are investigated. Second, CRLD simplifies the highly stochastic and computationally intense reinforcement learning algorithms into deterministic (differential or difference) learning equations. The connection between learning algorithms and equations either stems from stochastic approximation theory (51), or evolutionary game theory (28). The replicator dynamics from mathematical biology describe not only evolutionary processes but also individual learning processes (56). The relationship between the two fields is as follows: one population with a distribution over phenotypes in the evolutionary setting corresponds to one agent with a distribution over actions in the learning setting (57). Crucially, unlike other dynamical system methods that describe the macroscopic behavior of many agents in a low-dimensional dynamical system, CRLD can also describe the idealized learning behavior (58) of a single or few reinforcement learning agents.

What is still missing is an integrated theory of CRLD (Box 3), which explains from what principles different individual learning schemes arise, how they are related, and which algorithmic details matter on the collective level. Such a CRLD theory would enable us to summarize the idealized learning behavior of a broad set of algorithms. See refs. 51, 59, and 60 for promising starting points.

Box 3. Open research strands.

The following open research areas are intended to cross-fertilize progress on collective cooperative intelligence between CSS and MARL:

1. **CRLD Theory.** What are the principles that give rise to different RL update schemes? How are they related? Which algorithmic details matter on the collective level and which do not?
2. **Complex dynamic phenomena.** What are the conditions for the occurrence of and the potential use-cases for complex emergent and transient phenomena, such as multistability, abrupt transitions, hysteresis, and dynamic regimes in CRLD and large-scale MARL?
3. **CRLD with cognition.** How can cognitive mechanisms such as representational learning, world models, intrinsic motivations, and theory of mind be integrated into CRLD? What are their effects on the learning of cooperation? What is the role of intrinsic noise in CRLD?
4. **CRLD in large collectives.** What are the assumptions that result in mean-field approaches to MARL? How do they relate to each other and to CRLD? And what are the principles in CRLD from which cooperation can emerge in large collectives composed of intelligent individuals, particularly heterogeneous ones? How can these principles be transferred to large-scale MARL systems?
5. **CRLD in dynamic environments.** How do environments of different levels of abstraction relate to each other? What techniques are suitable for scaling environments up and down? Which environmental properties promote or hinder the learning of cooperation? How do these properties influence large-scale MARL systems?

### Complex Phenomena.

Statistical mechanics provides a formal framework to derive emergent phenomena from microscopic rules for systems that are too complex to analyze directly (61). The critical advantage of CRLD is the ability to uncover complex emergent phenomena within a MARL system in a computationally fast and lightweight manner. In this section, we highlight how a CSS perspective enriches the study of cooperation among intelligent agents in complex environments in MARL, using the framework outlined in Box 1 and paving the way toward a “strategic statistical mechanics” (62).

#### Multistability.

MARL studies typically report learning success over time. CSS aims to analyze the whole dynamics emerging from agents learning in the environment. This can be done by visualizing (projections of) the joint strategy space. For example, Fig. 1*A* shows the phase space in the prosperous state for the environmental example of Box 2 with the exemplary learning dynamics of Box 1. Each arrow represents the average effective reward-prediction error the agents perceive at this strategy point. They, therefore, give an intuition about the direction in which the collective will learn.

A phase space perspective highlights the stability landscape, e.g., indicating whether the current situation exhibits more than one equilibrium. For example, the blue learning trajectories in Fig. 1*A* enter the mutual-cooperation strategy equilibrium point. The red learning trajectories go to mutual defection. Assuming an equal likelihood of initial strategies, the size of the so-called basin of attraction for a given equilibrium indicates how likely the equilibrium will occur.

Multistability in collective learning has exciting applications. First, the size of the basin of attraction of the mutually cooperative solution is a valuable measure of collective (cooperative) intelligence (63). Second, stability landscapes and basins of attraction are instrumental to the study of social–ecological resilience (64), which make CRLD an ideal candidate to advance this field (65). Third, entering one over other possible equilibria is a form of storing information and thus can be seen as a form of emergent collective memory (66). This perspective can explain social conventions like technical standards, cultural norms, and moral rules. Applying it to engineering MARL systems offers the potential to outsource cognitive functions onto the collective. Fourth and last, the relative size of attraction basins offers a valuable perspective for ad hoc teamwork (67). If provides a geometric view of how new agents impact equilibria selection in groups of agents trained a priori when new agents—possibly with strategies hardcoded in a particular way (68)—are introduced in the system.

Around the basin of attraction’s edge, also known as “separatrix” (dashed purple line in Fig. 1*A*), the transient dynamics before entering equilibria show complex phenomena. For example, the fine-grained bundle of learning trajectories shows that, at the separatrix, only a tiny difference in the first agent’s initial cooperativeness is decisive for where both agents will end up (Fig. 1*B*). Near the bifurcation point, the learning dynamics may separate into fast and slow directions. Fig. 1*C* shows the two closest learning trajectories to the bifurcation. Both appear to have converged after ≈180 time steps and remain stable for over 200 time steps until they head off to drastically different strategies. This phenomenon occurs due to the underlying geometry of the strategy space being a so-called saddle node (purple cross in Fig. 1*A*). At the unstable saddle-node equilibrium, there are stable and unstable directions in strategy space. Along the stable directions, learning evolves on a slower timescale toward the saddle point. Once the agents are “past” the (unstable) equilibrium and enter the unstable directions, they diverge.

Finally, CRLD’s overall convergence time also depends on the initial strategies. Close to the separatrix, the convergence time is up to an order of magnitude larger than far away from it in our example (Fig. 1*D*). This phenomenon is known as critical slowing down, describing the increase in the system’s typical timescale close to a critical point. Here, the concept of convergence becomes less relevant, and a careful investigation of the transient learning dynamics is vital—especially when external parameters change and transitions between stable equilibria occur.

#### Critical transitions.

When small changes in external parameters have significant consequences for the system’s behavior, CSS speaks of a critical transition. Other terms for this phenomenon are regime shifts, bifurcations, tipping elements, or phase transitions. Some CRLD studies have already investigated such critical transitions in collective learning (53, 55, 69–72). External parameters in the MARL framework consist of the learning hyperparameters and the parameters defining the environment. Hyperparameters denote the parameters of the learning process, as opposed to parameters used to encode an agent’s strategy. An example of an agent’s hyperparameter is its learning rate, which determines the extent newly obtained information overrides old information. Parameters defining the environment shown in Box 2 are the severity and likelihood of collapse, *m*, and $q_{c}$. The analogy to phase transitions from classical statistical mechanics is as follows: a system (such as water) can exist in multiple stable forms (liquid, ice, steam) depending on external parameters (such as pressure and temperature). For standard room pressure, the transitions between these stable forms happen around the critical temperature points of 0 and 100 ^°^C. Fig. 2*A* shows the transition from complete defection to full cooperation by varying the agents’ discount factor, denoting how much the agents care for their future wellbeing. For discount factors below 0.7, full defection is globally stable (i.e., reached for any initial condition), and for those above 0.85, full cooperation is globally stable. In between, they are in a bistable regime where both full cooperation and full defection are stable (as in Fig. 1*A*). This can be seen by the abrupt change in the quartiles in Fig. 2*A*, whereas the smooth change in the average indicates a continuously shifting basin of attraction from full defection to full cooperation.

Near the critical region, the phenomenon of critical slowing down can be observed again. For discount factors of around 0.75, learning takes approximately twice as long as for small discount factors and about an order of magnitude longer than for high discount factors (Fig. 2*B*). While this phenomenon emphasizes the need to focus more on the transient learning dynamics, it also offers a potential application. So-called “early warning indicators” from CSS (73) could be utilized in collective learning processes to detect and proactively address nearby transition points.

Overall, the occurrence of critical transitions in multiagent learning makes CRLD a promising modeling tool for studying emergent social tipping points in social–ecological systems and human–environment interactions.

#### Hysteresis.

If we revert the external parameter after a critical transition, but the system does not follow the same trajectory back, we observe the phenomenon known as “hysteresis” (74–77). Fig. 3 shows hysteresis in CRLD. In contrast to Fig. 2, where the CRLD system was simulated to convergence for each parameter point independently, in Fig. 3, the external parameter (here, the discount factor) changes slowly within the simulation. Around a discount factor of 0.83, the CRLD system tips from defection to cooperation. It remains there until the discount factor decreases again until 0.7 when it tips again back to full defection. For discount factors between 0.7 and 0.83, the agents’ strategy depends on where the discount factor was before. More complex phenomena might arise when the timescales of external parameter changes approach the internal timescale of the learning dynamics (78).

Hysteresis means that the system’s state is dependent on its history of external parameter changes, i.e., it can store information. Thus, hysteresis in MARL presents another form of collective memory. Future work on (large-scale) MARL systems can benefit from more explicitly considering the complex networks between agents to outsource cognitive functions between the agents. For instance, in the line of work on autocurricula, a changing distribution of environments might endow (collectives of) agents with (cooperative) skills that robustly persist even as the distribution of environments changes over the course of learning.

Taken together, the complex emergent phenomena around multistability, critical transitions and hysteresis plus a plurality of possible dynamic regimes as shown in the *SI Appendix* in CSS offer great potential for analyzing and designing MARL systems. These phenomena are observable in CRLD, making it highly likely that they can also be found within high-dimensional MARL systems. However, it is still an open research question to systematically investigate the conditions for the occurrence and the potential use cases for emergent phenomena in MARL (Box 3).

### Cognition in Contexts.

Last, we discuss how ideas from MARL can enrich CSS regarding the three challenge areas of individual cognition, collective behavior, and dynamic environments.

#### Cooperation from individual cognition.

First, we explore how cooperation might emerge from individual cognition and what is needed to move CRLD to a position able to investigate this question.

CSS’s dominant interpretation of how successful strategies spread is by agents copying the strategies of more successful agents. This approach has been very influential in capturing the key elements of cultural evolution (79). However, this form of social learning assumes that agents share the same success criteria and can observe both strategies and the success of others, making it difficult to apply to asymmetric interactions. There is clear interest within CSS in extending this paradigm to other forms of cognition and learning (32, 80). The various cognitive mechanisms explored in CSS do not explain how to integrate them into one coherent model. Such integration, however, is necessary to examine their interplay and relative importance for emerging collective cooperation in dynamic environments. Moreover, cognition is general-purpose and must, therefore, be examined across different environments.

MARL offers a comprehensive framework for studying the interplay among learning, representation, and decision-making between multiple actors (81), offering an integrating platform to test hypotheses on how different cognitive mechanisms may affect collective cooperation in dynamic environments. For example, intrinsic motivations guide learning without relying on externally supplied rewards for improved exploration, control, and homeostasis (82). Being explicitly prosocial and including the rewards of others in one’s own reward function (i.e., having other-regarding preferences) clearly promotes cooperation (83). In general, however, it is nonobvious how other common types of intrinsic motivations affect cooperation, e.g., being curious (84), being cautious or risk-taking (85), being controlling (86), or being predictive (87). Even less is known about how other cognitive functions affect collective cooperation in dynamic environments. For example, a key component in single-player deep RL is experience-replay, bearing some resemblance to the replay events observed in the hippocampus (88). Other cognitive mechanisms are employed to learn efficient representations of high-dimensional observations (89) and internal models of the world (90). These mechanisms are crucial for agents to update their behavior in a robust and efficient way.

The benefit of CRLD is that it combines the integrating platform of classical MARL for testing hypotheses regarding cognition (Box 1) with the analysis toolbox of CSS. Deterministic CRLD model agents learn in the theoretical limit of an infinite memory batch (91) or as if they have a perfect model of the current environment (58). It is such abstractions that make these models useful. By comparing the deterministic CRLD dynamics with the stochastic MARL simulations, it has been shown that the intrinsic stochasticity of the MARL process is highly beneficial for the learning of cooperation (92). Lenient CRLD dynamics make agents forgiving to initial mismatched teammate actions, which result in a higher likelihood of converging to the cooperative solution. CRLD with partial observability can learn to cooperate through inaccurate representations of the environment (93). However, future research is needed to examine the role of more refined notions of cognition, such as adaptive intrinsic representations, world models, motivations, and theory of mind in the learning of cooperation, in general, and of intrinsic noise in the emergence of cooperation in dynamic environments by means of stochastic dynamical systems (94), in particular (Box 3).

#### Cooperation in large collectives.

Next, we discuss the challenge of cooperation in large collectives of self-learning agents in dynamic environments and how CRLD can investigate this problem.

In CSS, the canonical approach assumes a large collective of homogeneous and/or simple individuals, and there is a long tradition of describing the phenomena emerging from such collectives (28). For example, the famous replicator equation describes the evolutionary dynamics of a population containing an infinite number of individuals (95) and many works have investigated the preconditions for cooperation under this paradigm. Refining this paradigm transforms evolutionary dynamics from infinite to finite (but still large) populations (96).

In MARL, large collectives are much harder to study because of the exponentially increasing joint state-action space and the associated computational demands that come with it. However, some notable examples exist (97, 98). They manage the computational demands by careful software engineering, exploiting agent similarities through parameter sharing or using transfer learning mechanisms while progressively increasing the number of agents. Yet, little is known about cooperation in large collectives from a MARL perspective.

In CRLD, a few works have begun to explicitly treat a collective of RL agents as a nonlinear dynamical system, deriving a Fokker–Planck equation to describe the idealized learning behavior of a MARL collective. They focus on repeated symmetric games (49), population games (99), stochastic effects and incomplete information (100), regret minimization (101), and regular social network structures (102). There is an apparent similarity here with so-called mean-field approaches to MARL. MARL is often very high-dimensional and highly stochastic, making it computationally challenging and difficult to understand. Mean-field approaches, originally from theoretical physics, attempt to solve this problem by approximating the original system with a simpler model by averaging over certain degrees of freedom. It is, therefore, an ideal tool for gaining conceptual clarity about collective cooperation in complex settings. However, being a research frontier, mean-field approaches to RL are not yet consolidated, and different variants exist. Most prominently, mean-field games (103) consider the large-agent limit in a situation where each agent has an individual state associated with it. Other works consider the mean action of a local neighborhood of agents (104), which allows the study of a finite number of agents. All variants primarily focus on obtaining convergence guarantees. Future work is needed to distill the emergent phenomena, transient dynamics, and principles that lie behind cooperation in large collectives composed of intelligent individuals, particularly heterogeneous ones from a CRLD perspective. To do so, it will be vital to consolidate these different mean-field approaches, contrast the assumptions from which these arise, and refine them to heterogeneous and structured populations (Box 3).

#### Cooperation in dynamic environments.

Last, we explore the challenge of cooperation in dynamic, stateful environments and what is required to leverage the most out of the CRLD approach.

In CSS, recent years have seen a growing interest in moving from evolutionary dynamics in stateless games to dynamic environments. Here, the term “dynamic” can mean external fluctuations (105), a varying population density (106), spatial network structure (107), or coupled systems of evolutionary and environmental dynamics. Coupled systems may be categorized into those with continuous environmental state spaces (108) or discrete ones (109). These examples reflect a diverse understanding of the term “environment.” However, agents typically remain in the paradigm of social learning in these works.

The MARL setting employs large, dynamic, and uncertain environments via the framework of (partially observable) stochastic games. Sequential social dilemmas form a bridge between high-dimensional environments and the simple, stateless games primarily used to study cooperation in CSS (39, 83). They share simple games’ mixed incentive structure but require agents to learn cooperation strategies on their own, i.e., cooperation is not a single atomic choice but a temporally extended sequence of actions. See *SI Appendix* on how to define “cooperation” in stateful environments. For such complex environments, deep MARL can also be used to generate predictions of human behavior, where traditional game-theoretic methods are no longer amenable (110).

Some CRLD works already consider dynamic environments via learning in (partially observable) stochastic games (44, 111). To leverage their strengths in abstraction, CRLD studies should focus on comparably low-dimensional stochastic games with few environmental states to combine the best of both worlds: analytical tractability and environmental complexity. For example, our guiding example (Box 2) is a stochastic game with only two states. Nevertheless, it is sufficient to highlight a range of complex phenomena (see above) and calculate analytically when the level of caring for future rewards can turn the tragedy of the commons into a comedy—where cooperation prevails—without requiring any form of social reciprocity (91). Future work is required to explore more generally which environmental conditions favor or hinder the learning of cooperation (Box 3). Factors to consider are environmental uncertainty, delayed and sparse rewards, the coupling of local and global environmental states, environmental tipping points, and spatial extendedness. To do so, a better understanding of how environments on different abstraction levels are related. Empirical Game Theoretic Analysis downscales a high-dimensional environment to a stateless game via heuristic strategies (112). Intuitively, this technique complements the sequential social dilemma concept (39), reducing an environmentally complex situation to its core. Future work should aim to downscale a high-dimensional environment not necessarily to a single environmental state but to a few significant states to preserve the essential environmental context (see also *SI Appendix*). For this purpose, conceptual links to temporally extended substrategies of hierarchical reinforcement learning may be fruitful (113).

## Conclusions

Collective cooperation—in which intelligent actors in complex environments seek ways to improve their joint well-being—is critical for a sustainable future, yet unresolved. Mathematical models are essential to solve this challenge. Here, we argue that building bridges between CSS and MARL offers a more robust understanding of the drivers, mechanisms, and dynamics of collective cooperation from intelligent actors in dynamic environments. Both fields complement each other in their goals, methods, and scope.

CSS offers MARL improved qualitative insight into emergent collective learning dynamics via its analysis toolbox. Based on the existing body of works that models MARL as nonlinear dynamical systems, we highlighted what kinds of insights a CSS perspective on complex, emergent phenomena and dynamics offers. In our guiding example (Box 2), all three elements of cognition, collective, and environment are represented in their most minimal form.

By way of return, MARL offers CSS concrete ways to formalize cognitive processes in dynamic environments. We portrayed the current state of cooperation research along the three elements of cognition, large collectives, and dynamic environments. Box 3 summarizes the five open research strands we identified. Ultimately, by sharing the same mathematical framework, MARL and CSS complement each other in their scope. CSS can focus on more qualitative, conceptual models with comparable low-dimensional environments. In contrast, MARL can focus on scaling cooperation-promoting principles to more quantitative, realistic models with comparable high-dimensional environments.

This complementary view of MARL and CSS can also strengthen the role of theory. It enables the formulation of integrated theories around the dynamics of cooperation in large collectives from individual cognition in environmental contexts. The connection between empirical data is threefold. 1) On the behavioral level, theory predictions can be compared with behavioral experiments or field observations. In addition to procedures with human or nonhuman animal subjects, large language models (LLMs) offer innovative ways to investigate various phenomena in human-machine ecologies. 2) On the neurological level, stylized theoretical representations of neural information processing could be compared with natural or artificial brain data (as obtained, e.g., in neuroeconomics). 3) Biophysical and socioeconomic data can be used to parameterize the model environment. The resulting learning dynamics offer projections of future socioeconomic pathways, and the distance to critical points in parameter space gives a measure of their resilience.

We believe that the approach of building bridges between CSS and MARL provides the necessary foundations for a genuine science of collective, cooperative intelligence and invite everyone to join forces.

## Supplementary Material

Appendix 01 (PDF)

## Data Availability

All supplementary information in fully reproducible form is available here (114) or in the *SI Appendix*.
