# Supplementary material for: Collective cooperative intelligence
Source: Proc Natl Acad Sci U S A. 2025 Jun 16;122(25):e2319948121. doi: 10.1073/pnas.2319948121 (PMC12207418; doi:10.1073/pnas.2319948121)
Supplement: Supplementary file 1 — Appendix 01 (PDF) [file pnas.2319948121.sapp.pdf]

Supplementary Information for Collective Cooperative Intelligence

Wolfram Barfuss et al.

June 12, 2024

Table of contents

1 Introduction **3**

2 Literature background **4**
2.1 Complex Systems Science (CSS) 4
2.2 Multi-Agent Reinforcement Learning (MARL) 6
2.3 Examplary works on the learning dynamics of cooperation 7
2.4 On cooperation and social dilemmas 8

3 Framework **11**
3.1 Multi-agent environment interface (MAEi) 11
3.2 Ecological Tipping Environment 12
3.3 Reinforcement learning 14
3.4 Collective Reinforcement Learning Dynamics (CRLD) 16

4 Multi-stability **20**
4.1 Phase space plot 20
4.2 Sample trajectories 22
4.3 Critical slowing down 23
4.4 Detailed phase space 24
4.5 Time scale separation 25

5 Abrupt transitions **27**
5.1 Compute data 27
5.2 Plotting function 28
5.3 Abrupt transition 29
5.4 Critical slowing down 31

6 Hysteresis **33**
6.1 Compute data 33
6.2 Plot curve 34

7 Dynamic regimes **36**
7.1 Memory-one prisoners' dilemma environment 36
7.2 Compute data 38
7.3 Plot data 39
7.4 Find initial strategy 40

8 Simulation Scripts **43**
8.1 Create initial strategies 43
8.2 Compute trajectories 44
8.3 Saving & reloading 45
8.4 Final rewards 46

9 References **47**

1 Introduction

Collective cooperation – in which intelligent actors in complex environments seek ways to improve their joint well-being – is critical for a sustainable future, yet unresolved. Mathematical models are essential for moving forward with this challenge. Our perspective paper argues that building bridges between CSS and MARL offers a more robust understanding of the drivers, mechanisms, and dynamics of collective cooperation from intelligent actors in dynamic environments. Both fields complement each other in their goals, methods, and scope.

This supplementary information presents a more detailed background on the literature (Chapter 2). Furthermore, we give all the details regarding the collective reinforcement learning dynamics we employ (Chapter 3) and how we applied it to create all complex phenomena presented in the main text (Chapter 4 - Chapter 7). Chapter 8 contains all required simulation scripts.

Reproducibility

This supplementary information was created in a *fully reproducible writing and computing environment* with the help of nbdev and quarto. If you are reading the PDF or web version of this document, you can find the source code in form of Jupyter notebooks at https://github.com/wbarfuss/collective-cooperative-intelligence.

To reproduce all simulations, create a new conda environment with the provided `pythonenvironment.yml` file.

```
conda env create -f pythonenvironment.yml
```

This installs also the Collective Reinforcement Learning Dynamics in Python. They are provided by a separate Python package, which is in its early stages of development.

You activate the environment with:

```
conda activate cocoin
```

Afterwards, you should be able to follow along and execute all notebooks.

If you have any feedback, questions or problems with code, please do not hesitate to open a Github issue here: https://github.com/wbarfuss/collective-cooperative-intelligence/issues.

2 Literature background

2.1 Complex Systems Science (CSS)

Complex systems are generally out of equilibrium with many interacting components, feedback, and couplings between components and levels (e.g., Levin, 2002). Emergent, collective behavior at the macro level is surprising and hard to predict. An extensive repertoire of strategies and interaction types at the component level makes for multiple emergent patterns and functions at the macroscale–the state of the art to date largely treats each pattern independently. Consequently, there is little understanding of the overall degree of collectivity (Daniels et al., 2021). Power law and heavy-tailed distributions can lead to consequential 'black swan' and second and third-order effects (De Marzo et al., 2022). Some complex systems sit near a critical point at which small perturbations can cause a phase transition or reconfiguration because of long-range correlations (Mora & Bialek, 2011), including in finite, relatively small systems like many animal societies and human groups (Daniels et al., 2017).

CSS methods are diverse. Those most relevant to understanding emergence of cooperative collectives include nonlinear dynamics to study temporal oscillations and couplings in time such as how individuals synchronize their activities (Sarfati et al., 2021), stochastic differential equations to study how, for example, noise influences transitions between disordered and ordered states (Jhawar et al., 2020), approaches from statistical mechanics to study collective behavior in space, such as how swarms (Buhl et al., 2006) and flocks choose trajectories (Bialek et al., 2012), game theory (Hofbauer & Sigmund, 1998; Nowak, 2006), network theory (Newman, 2003) to quantify interaction structure and how for example individuals make decisions under the influence of others in uncertain environments (Kleshnina et al., 2023), cellular automata (Wolfram, 1994) to gain insight from toy models about the relationship between rule complexity and pattern formation, agent based modeling (Epstein & Axtell, 1996), the physics of information to identify the mechanisms supporting information processing and quantify their efficiency with the goal of understanding how energy and information processing interact to shape collective effects (Kempes et al., 2017), and information theory to identify and quantify the contribution of higher order interactions to macroscale effects (Rosas et al., 2019; Tekin et al., 2017, 2018), quantify overall degree of collectivity (Daniels et al., 2016), and to build unifying frameworks leveraging ideas from predictive coding (Darriba & Waszak, 2018; Friston, 2018; Rao & Ballard, 1999), active inference and the free-energy principle (Buckley et al., 2017), and the information theory of individuality (D. Krakauer et al., 2020) for formalizing the role of uncertainty reduction (also called surprise minimization (Heins et al., 2023)) in micro-macro relationships and entity formation and evolution.

We expect information theoretic approaches emphasizing uncertainty reduction will be particularly productive for informing the development of a 'strategic statistical mechanics' that brings together powerful probabilistic approaches from statistical physics and information theory for deriving micro-macro maps with logical principles from theoretical computer science and the study of inference to capture robust and optimal design of how strategies interact in social circuits to support cooperation at scale. For example, a recent paper on surprise minimization (Heins et al., 2023) makes substantial progress in this direction. The authors develop a modeling framework to capture spatial collective behavior with inference-capable agents. The agents can estimate hidden causes of their sensations and adapt their position in space to minimize surprise (prediction error). The authors then study the relationship between individual inference and the emergence of collective states like cohesion and milling. Next steps include 1) exploring the pros and cons of active inference vs. MARL for encoding

cognition into agents, 2) combining this approach with inductive game theory (probabilistic strategies are empirically grounded and extracted from time series) (DeDeo et al., 2010; D. C. Krakauer et al., 2010), and 3) studying how collective states and their transitions are computed from the social circuits (Brush et al., 2018; DeDeo et al., 2010; Ramos-Fernandez et al., 2020) that form as strategies are updated and consolidate into slow variables, which reduce social uncertainty and permit accelerated rates of adaptation (Flack, 2017). With these extensions, it should be possible to begin to deduce the organizational and algorithmic principles underlying the emergence of micro-macro maps in collective information processing systems through feed-forward effects and downward causation. These principles will likely inform the conditions under which collective, cooperative intelligence emerges at scale.

It is worth noting that in the models developed in the surprise minimization, inductive game theory, and some of the collective computation work described above, individuals are tracked, making these models, in a sense, agent-based models. However, these approaches distinguish themselves by incorporating agents that model the world in a restricted but cognitively principled manner or by parameterizing the models using probabilistic strategies obtained directly from data and by leveraging the rigor of powerful probabilistic modeling frameworks in statistical physics or dynamical systems traditions. In the more conventional agent-based modeling community, there have also been attempts to develop a more rigorous axiomatic approach, e.g., based on symmetries and bifurcation theory (Franci et al., 2022; Park et al., 2021) and game theory and control (Marden & Shamma, 2018).

Work within the game theory and cultural evolution CSS sub-communities has made strides in understanding the social and cultural dynamics resulting from interacting boundedly rational agents with a finite computational budget. This work focuses on social and cultural learning mechanisms that allow agents to improve their behavior over time (Arthur, 1994, 2014; Holland & Miller, 1991). Game theoretic models in this tradition aim to explain emerging cooperation from simple yet plausible mechanisms. For example, the famous strategy tit-for-tat, which merely reciprocates what the opponent did in the previous turn, is surprisingly successful against much more complicated strategies (Axelrod & Hamilton, 1981). Its success can be attributed to its ability to control payoffs, ensuring that it receives the same score as the opponent, regardless of the complexity of the opponent's strategy. The "zero-determinant" strategies later discovered by Press & Dyson (2012) provide a vast generalization of this phenomenon, allowing for extortion and generosity, in addition to more equitable relationships like that of tit-for-tat. These strategies have also encouraged a more geometric view of behavior in CSS (Hilbe, Chatterjee, et al., 2018), moving away from purely mechanistic descriptions.

An unsatisfactory facet of many of these game theoretic and cultural and social evolution models is that "cooperation" is based on an atomic action with the property that more cooperation translates to better social welfare. This interchangeability is likely part of the reason for the widespread focus on mechanisms for increasing the level of cooperation within a system. However, even for the most basic model of a conflict of interest, the repeated Prisoner's Dilemma, it can be the case that high levels of "cooperation" are suboptimal for individuals and the collective, e.g., when agents are better off alternating "cooperation" and "defection" over time (relative to always cooperating) (McAvoy et al., 2022). Along these lines, nonlinearities produce counter-intuitive or hard-to-predict dynamics, meaning that it is essential to consider not only the level of cooperation but also the specific collective states or social outcomes that emerge from alternative strategic configurations and game structures. With their simplifying assumptions, these approaches are suitable for gaining insight into null expectations for baseline conditions but are more limited in utility when tackling cooperation at scale in complex environments composed of cognitively complex, error-prone agents (McNamara, 2013).

Humans routinely deviate from the behavior predicted by the economic model of *Homo economicus* (Camerer, 2011). Yet, they are also more sophisticated than assumed in many simple evolutionary game theory models. They are capable of foresight, have a theory of mind, make inferences about their environment, and can adapt their behavior correspondingly. For example, in the most common evolutionary game theory models, individuals from a large population are randomly matched with other population members to play a static game. Those individuals who are more successful (because

they employ better strategies) are more likely to be imitated. Imitation-based models are most appropriate when interactions are symmetric in the sense that individuals coincide in their feasible actions and payoffs. However, the paradigm is more challenging to motivate among heterogeneous and diverse actors and when behaviors cannot be observed directly and must be inferred before they are imitated. Moreover, extending this paradigm to account for other forms of cognition that intelligent individuals typically employ when revising their strategies is not straightforward. Finally, the act of imitation itself may be learned and, therefore, subject to cognitive constraints and learning dynamics (Team et al., 2022).

There is a clear interest in adapting game theoretic and cultural evolution models to accommodate these nuances (Hauser et al., 2019; Hilbe, Šimsa, et al., 2018; McNamara et al., 2021; X. Wang & Fu, 2020). As with the uncertainty reduction and collective computation approaches discussed above, considering how MARL could inform such models has great potential to unleash novel ways of modeling complex systems to tackle the challenges of collective cooperation in more complex settings.

2.2 Multi-Agent Reinforcement Learning (MARL)

In a typical MARL setting, each agent observes (part of) the current state of the environment, then takes an action, after which they observe (part of) the new state of the environment and are provided with a reward indicating how desirable the previous “state-action-state” transition was. Over time, the agents update their strategies (a mapping from observation histories to probability distributions over their action space) to increase the long-term amount of reward they receive (Busoniu et al., 2008). In this work, we employ a broad definition of reinforcement learning (RL), including various individual-based update mechanisms. However, we exclude strategy update processes based on social reward comparisons, such as typical evolution models and explicit social learning. Eventually, we are interested in how processes, such as social learning, opinion formation, and collective action, can emerge from individual learning agents.

Modern MARL is inspired by work in several fields, including neuroscience, psychology, economics, and machine learning (Bush & Mosteller, 1951; Cross, 1973; Dayan & Niv, 2008; Erev & Roth, 1998; Fudenberg & Levine, 1998; Roth & Erev, 1995; Sutton & Barto, 2018). For example, the commonly used idea of temporal-difference learning is based upon reward-prediction errors, common to humans, other animals, and machines (Botvinick et al., 2020; Gunawardena, 2022; Schultz et al., 1997). In recent years, these traditional ideas have been combined with advances in machine learning – in particular, deep learning – to produce spectacular successes in various domains (Berner et al., 2019; Silver et al., 2016; Vinyals et al., 2019).

Studies of cooperation in MARL fall under the umbrella of *Cooperative AI* (Dafoe et al., 2021). They can be divided based on whether the underlying game is fully cooperative (i.e., where all agents share the same goal) or mixed-sum (as opposed to zero-sum, which describes fully competitive situations). MARL as a field does not have a unique goal (Shoham et al., 2007). For example, some works aim to obtain game-theoretic equilibria via MARL, while others ask which learning rules are in equilibrium with one another in a specific environment. Despite this variety, the overarching aim of Cooperative AI is to improve the cooperative capabilities of AI systems, increasing joint welfare by prescribing how agents should (learn to) act. Such learning algorithms should ideally generalize to novel situations and scale to high-dimensional environments. A vital advantage of the MARL paradigm is that it can easily accommodate heterogeneous actors. Extending machine learning interpretability techniques to MARL is an ongoing effort to advance the understanding of MARL systems (Grupen et al., 2022; Lovering et al., 2022; McGrath et al., 2022).

Methodologically, the focus often lies in designing novel algorithmic features to improve the cooperativeness of RL algorithms in large-scale environments. For example, algorithms may be equipped with

abilities, such as sending each other messages (Foerster et al., 2016), making commitments (Christoffersen et al., 2022; Hughes et al., 2020), or transferring rewards to others (Lupu & Precup, 2020; W. Z. Wang et al., 2021). Algorithms are evaluated for their ability to produce agents and multi-agent systems that can generalize, i.e., perform well under conditions they never saw during training, such as situations where they must interact with unfamiliar AI social partners (Leibo et al., 2021; Stone et al., 2010) or humans (Carroll et al., 2019; (FAIR)† et al., 2022; Strouse et al., 2021). Measuring generalization to a fixed set of test scenarios allows researchers to compare the performance of MARL algorithms to one another despite incompatibilities in their training. In contrast to CSS studies, *cooperation* is typically not an available action to choose from. Instead, implementing a cooperative strategy must be learned from scratch (Leibo et al., 2017), and performance is measured by total social welfare.

However, MARL simulation studies on their own are challenging to use to obtain analytically reliable insights into how collective cooperation emerges from complex human and machine behavior in dynamic environments. They often require significant computational resources, while the space to explore suffers from the curse of dimensionality. Moreover, they are typically highly stochastic, and results can be difficult to interpret (Hernandez-Leal et al., 2019). We believe that a unified approach that combines approaches from CSS and MARL could fill this gap.

2.3 Examplary works on the learning dynamics of cooperation

The study of cooperation has not been at the center of Collective Reinforcement Learning Dynamics (CRLD) studies. Here we list some notable examples from mathematical biology and sociology.

- L. Panait, K. Tuyls, S. Luke, Theoretical advantages of lenient learners: An evolutionary game theoretic perspective. J. Mach. Learn. Res. 9, 423–457 (2008).
- S. S. Izquierdo, L. R. Izquierdo, N. M. Gotts, Reinforcement learning dynamics in social dilemmas. J. Artif. Soc. Soc. Simul. 11, 1 (2008).
- M. Wunder, M. L. Littman, M. Babes, Classes of multiagent Q-learning dynamics with epsilon-greedy exploration in ICML (2010).
- N. Masuda, M. Nakamura, Numerical analysis of a reinforcement learning model with the dynamic aspiration level in the iterated Prisoner's dilemma. J. Theor. Biol. 278, 55–62 (2011).
- S. Tanabe, N. Masuda, Evolution of cooperation facilitated by reinforcement learning with adaptive aspiration levels. J. Theor. Biol. 293, 151–160 (2012).
- T. Ezaki, Y. Horita, M. Takezawa, N. Masuda, Reinforcement learning explains conditional cooperation and its moody cousin. PLoS Comput. Biol. 12, e1005034 (2016).
- S. Dridi, E. Akçay, Learning to cooperate: The evolution of social rewards in repeated interactions. Am. Nat. 191, 58–73 (2018).
- O. Leimar, J. M. McNamara, Learning leads to bounded rationality and the evolution of cognitive bias in public goods games. Sci. Rep. 9, 16319 (2019).
- W. Barfuss, J. F. Donges, V. V. Vasconcelos, J. Kurths, S. A. Levin, Caring for the future can turn tragedy into comedy for long-term collective action under risk of collapse. Proc. Natl. Acad. Sci. U.S.A. 117, 12915– 12922 (2020).
- W. Z. Wang et al., "Emergent prosociality in multi-agent games through gifting" in Twenty-Ninth International Joint Conference on Artificial Intelligence (2021), vol. 1, pp. 434–442.
- L. Wang et al., Lévy noise promotes cooperation in the Prisoner's dilemma game with reinforcement learning. Nonlinear Dyn. 108, 1837–1845 (2022).
- W. Barfuss, J. M. Meylahn, Intrinsic fluctuations of reinforcement learning promote cooperation. Sci. Rep. 13, 1309 (2023).

2.4 On cooperation and social dilemmas

In CSS, cooperation is frequently defined mechanistically. A cooperative act might involve colluding with a co-conspirator to remain quiet under interrogation (Poundstone, 2011), paying a cost to provide a benefit to another (e.g., measured in currency, time, or reproductive success) (Sigmund, 2010), or provisioning a public good or resource (Fehr & Gächter, 2000; Ostrom et al., 1992). Game theory allows such a behavior to be modeled using abstract payoffs. Dawes (1980) summarizes a social dilemma among N agents, each with two atomic actions, C ('cooperate') or D ('defect'), as follows:

- *(i)* the payoff when all cooperate exceeds that when all defect and
- *(ii)* regardless of the composition of the group, a cooperator can always improve their own payoff by switching to defection.

A simple example is a prisoner's dilemma, which takes place in a collective of $N = 2$ agents. With payoffs defined by the matrix

$$\begin{array}{c|cc} & \text{C} & \text{D} \\ \hline \text{C} & R & S \\ \text{D} & T & P \end{array} \tag{2.1}$$

a social dilemma requires $T > R > P > S$, which is the *definition* of a prisoner's dilemma (Axelrod, 1984).

Cooperation becomes a graded quantity when a social dilemma is repeated, although it is still based on (atomic) cooperative actions in each round. As Leibo et al. (2017) note, what constitutes cooperation in spatially and/or temporally extended environments is more complicated and cannot determined using just reduction to a prisoner's dilemma via empirical game-theoretic analysis (EGTA). EGTA is an approach to game theory that combines expert modeling with empirical data of gameplay. High-dimensional game models are reduced to so-called meta-games via a small set of heuristic strategies. The meta-game, or empirical game, is a simplified model of the high-dimensional game that is used to gain an improved qualitative understanding of the complex multi-agent interaction (Tuyls et al., 2019).

Avoiding mechanistic considerations altogether, a useful way of thinking about cooperation is in terms of how a collective can jointly achieve higher payoffs, particularly when individual agents cannot force such outcomes. Suppose that the outcome $r^* \in \mathbb{R}^N$ is supported in Nash equilibrium. By the definition of a Nash equilibrium, no agent can improve its payoff through unilateral deviations in its policy. Therefore, if $r \in \mathbb{R}^N$ is another outcome for which $r_i \geqslant r_i^*$ for all $i = 1, \dots, N$, with at least one inequality strict, then no agent that would strictly benefit when the collective moves from r^* to r can force this outcome, even though all agents would be at least as well off in r as in r^*. Doing so is said to require 'cooperation' (Cohen, 1998).

Thinking of cooperation in this way hearkens back the notion of a social dilemma. If (D, D) is a Nash equilibrium, then $P > S$. Neither (C, D) nor (D, C) can Pareto-dominate (D, D) due to this inequality, so for 'cooperation' to exist it must be the case that $R > P$. One possibility for the final payoff is that $T \leqslant R$, in which case (C, C) is also a Nash equilibrium. Such is the case in the stag hunt game (Skyrms, 2004). Although this situation describes an interaction in which social welfare can be improved via cooperation, it is not strictly a social dilemma by the definition we used above, because the incentives of the individuals and the pair are not opposing. Rather, it represents an equilibrium selection problem. If, $T > R$ instead, then $T > R > P > S$, the defining inequalities of a prisoner's dilemma.

Importantly, the Pareto-dominated outcome (r^* above) need not be supported in Nash equilibrium in order to define a relevant notion of cooperation. Instead, one might impose the condition that there exists no sequence of unilateral, individually-rational deviations leading the outcome from r^* to an outcome that Pareto-dominates r^*. For the game depicted in Equation 2.1, this condition allows

$S \geqslant P$ as long as $T > R$. Such is the case in the snowdrift game (Sugden, 2004), in which two drivers are stuck on either side of a snowdrift blocking the road and must decide on who clears it. In contrast to the prisoner's dilemma, a driver is still better off cooperating (clearing the snowdrift) even when the other driver does nothing. One would also prefer to have the co-player do all of the clearing than to collaborate. A simple example in which 'cooperation' does not exist–even under this relaxed definition–is the harmony game (Hauert, 2002), which satisfies $R > T > S > P$ and possesses the property that the unique Nash equilbrium, (C, C), is also Pareto-efficient.

The prisoner's dilemma, and more generally the definition of Dawes (1980), characterize 'strict' social dilemmas. There are also 'weaker' social dilemmas describing conflicts of interest to lesser degrees. Again using the game in Equation 2.1, Hauert et al. (2006) stipulate that a weak social dilemma should satisfy

- *(i)* $R > P$;
- *(ii)* $T > S$; and
- *(iii)* $R > S$ and $T > P$.

The intuition behind these conditions is that *(i)* the payoff for mutual cooperation should exceed that of mutual defection; *(ii)* in mixed groups, the payoff to defectors should exceed that of cooperators; and *(iii)* regardless of what action a focal agent takes, they are better off when the co-player cooperates than when the co-player defects. The harmony game satisfies these inequalities, so it is considered a weak social dilemma despite the fact that it has no notion of 'cooperation' according to the definition of a 'strict' social dilemma. In addition to the prisoner's dilemma and the harmony game, the remaining two weak social dilemmas are the snowdrift and stag hunt games. As one might expect, the behavior of a weak social dilemma in CSS depends on which of these classes of games it falls under (Hauert & Doebeli, 2004), rather than just the fact that it's a weak social dilemma.

However, even in strict social dilemmas, we caution that the presence of alternative actions can destabilize conflicts of interest. For example, suppose that in addition to the actions C and D in a prisoner's dilemma, each player can take action G, which is interpreted as avoiding the prisoner's dilemma and instead collecting a pot of gold (at no cost). If both players have separate pots of gold available to collect and the value of this gold exceeds all of the prisoner's dilemma payoffs, then the unique Nash equilibrium of this augmented game is (G, G), which is also Pareto-efficient. Like in the harmony game, there is no strict notion of 'cooperation' in the sense of Pareto dominance. Most importantly, there is no conflict of interest and thus no strict social dilemma. It is irrelevant that there are options C and D such that $T > R > P > S$; this 'embedded' game is merely a decoy. Only when the action G is unavailable or unknown would the agents view this interaction as a social dilemma. In this sense, social dilemmas need not be preserved upon inclusion into larger games. In this example, one can easily recognize the option G as trivializing the game, but in realistic applications, especially those involving EGTA, it might be entirely unclear whether there are true conflicts of interest. Intriguingly, the augmented game described above could still be considered a sequential social dilemma (Leibo et al., 2017), owing to the fact that the reference policies representing cooperation and defection can be chosen freely (and thus can represent policies in a smaller, embedded game).

Along these lines, the reduction to matrix games via EGTA could result in too much averaging with respect to social dilemmas. One might instead map a stochastic game not to a matrix game but to a down-sampled stochastic game with a smaller number of 'salient' states. A simple example would be when two agents interact in a grid world, with two colors distributed throughout the grid according to some distribution. The two players drift throughout the space via independent, unbiased random walks. When they appear on neighboring tiles, they play one of two matrix games, a prisoner's dilemma or a harmony game, depending on whether the tiles have the same or different colors. There are then three relevant matrix games: the two played when on neighboring tiles and one 'null' game in which rewards are zero when neighbors on non-neighboring tiles. While one may view this game as having a large state space based on the agents' positions on the grid, this scenario can also be modeled as a three-state game whose transitions are governed by a hidden Markov model (due to the

structure of the grid and configuration of its colors). Nonetheless, by averaging appropriately, one might expect to obtain a useful approximation via a stochastic game with just three states. It is an open question whether further reduction to a matrix game would wash out important artifacts of this spatially-extended game.

Regarding cooperation and the goals of CSS, one (rather misleading) aspect of CSS models that could be informed better by the goals of MARL is the fact that social welfare, even in strict social dilemmas, need not be a monotonic function of the level of cooperation. The goal should not always be 'more cooperation' in a mechanistic sense. In the prisoner's dilemma, many studies in CSS make the simplifying assumption that $R > (S+T)/2$, which implies that a socially efficient outcome can be attained by full mutual cooperation. However, there are also prisoner's dilemmas for which $R < (S+T)/2$, in which case both agents can do better by agreeing on a strategy of alternation: one agent cooperates in even time steps only, while the other cooperates in odd time steps only. Moving from the mutually cooperative outcome of (R, R) to the Pareto-dominant outcome of $((S+T)/2, (S+T)/2)$ requires 'cooperation', despite the fact that the latter involves a lower level of the atomic action 'cooperate' than the former. Thus, what constitutes a cooperative strategy in a temporally extended social dilemma might be decoupled from what constitutes a cooperative action in the underlying stage game, an observation that has not fully penetrated CSS (McAvoy et al., 2022) despite being understood in MARL (Leibo et al., 2017).

In summary, what constitutes 'cooperation' depends on the context. In both CSS and MARL, seemingly isolated systems of agents can involve externalities that affect how an interaction is characterized/understood. If a cooperative social dilemma is actually a zero-sum game among N players and the environment, with the environment getting depleted as the social welfare increases, then the 'goals' in such an environment are ambiguous. Agents might also transition between such states and those involving the possibility of true surpluses. Complicating matters further, agents could transition among states involving different numbers of agents, including those with only a single agent and the environment. In turn, an agent can reasonably have many different conceptions of what 'cooperation' means, even on short timescales. Rolling such ephemeral interactions into 'cooperative strategies' is only more complicated.

3 Framework

3.1 Multi-agent environment interface (MAEi)

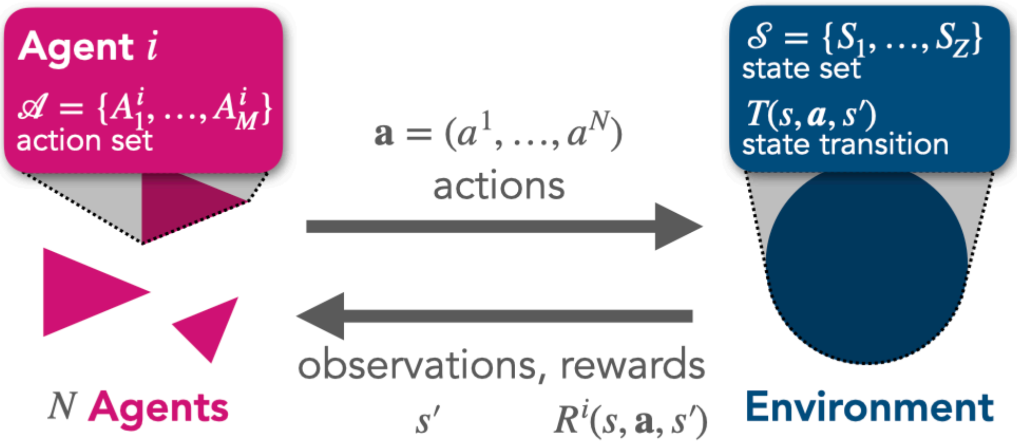

Figure 3.1: Multi-Agent Environment Interface (MAEi)

The basis for the learning dynamics is the multi-agent environment interface (MAEi) (Figure 3.1), which itself is based in its most basic form on the formal framework of stochastic games, also known as Markov games (Littman, 1994), which consist of the elements $\langle N, \mathcal{S}, \mathcal{A}, T, R \rangle$.

In an MAEi, $N \in \mathbb{N}$ agents reside in an environment of $Z \in \mathbb{N}$ states $\mathcal{S} = (S_1, \ldots, S_Z)$. In each state s, each agent $i \in \{1, \ldots, N\}$ has a maximum of $M \in \mathbb{N}$ available actions $\mathcal{A}^i = (A_1^i, \ldots, A_M^i)$ to choose from. $\mathcal{A} = \bigotimes_i \mathcal{A}^i$ is the joint-action set where $\bigotimes_i$ denotes the cartesian product over the sets indexed by i. Agents choose their actions simultaneously. A joint action is denoted by $a = (a^1, \ldots, a^N) \in \mathcal{A}$. With $a^{-i} = (a^1, \ldots, a^{i-1}, a^{i+1}, \ldots, a^N)$ we denote the joint action except agent i's, and we write the joint action in which agent i chooses a^i and all other agents choose a^{-i} as $a^i a^{-i}$. We chose an equal number of actions for all states and agents out of notational convenience.

The transition function $T : \mathcal{S} \times \mathcal{A} \times \mathcal{S} \to [0, 1]$ determines the probabilistic state change. $T(s, a, s')$ is the transition probability from current state s to next state s' under joint action a. Throughout this work, we restrict ourselves to ergodic environments without absorbing states.

The reward function $R : \mathcal{S} \times \mathcal{A} \times \mathcal{S} \to \mathbb{R}^N$ maps the triple of current state s, joint action a and next state s' to an immediate reward scalar for each agent. $R^i(s, a, s')$ is the reward agent i receives. Note that the reward function is often defined as depending only on the current state and joint action, $R^i(s, a)$. Our formulation maps onto this variant by averaging out the transition probabilities towards the next state according to $R^i(s, a) = \sum_{s'} T(s, a, s') R^i(s, a, s')$.

In principle, agents could condition their probabilities of choosing action on the entire history of past play. However, doing so is not only cognitively demanding. It also requires that agents observe all other agents' actions. Therefore, we focus our analysis on simple, so-called Markov strategies, with which agents choose their actions based only on the current state: $X^i : \mathcal{S}^i \times \mathcal{A}^i \to [0, 1]$. $X^i(s, a^i)$ is the probability that agent i chooses action a^i given the environment is in state s. We denote the joint strategy by $X = X(s, a) = \bigotimes_i X^i(s, a^i) : \mathcal{S} \times \mathcal{A} \to [0, 1]^N$.

3.2 Ecological Tipping Environment

We illustrate an application of the multi-agent environment interface by specifying a concrete environment that allows studying the prospects of collective action under environmental tipping elements (Barfuss et al., 2020).

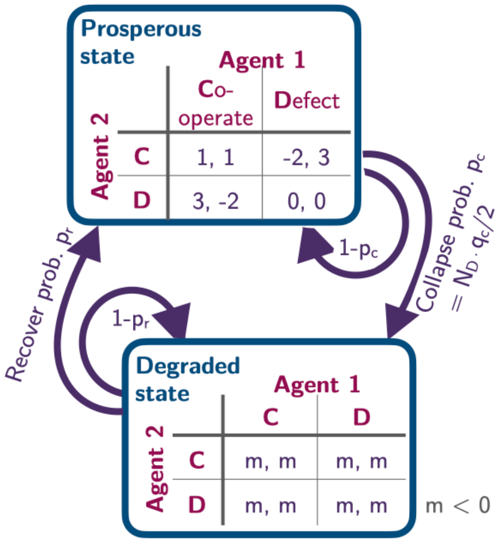

Figure 3.2: Ecological Tipping Environment

It is available in the Python package via:

```
from pyCRLD.Environments.EcologicalPublicGood import EcologicalPublicGood as EPG
env = EPG(N=2, f=1.2, c=5, m=-5, qc=0.2, qr=0.01)
```

The environmental state set consists of two states, a prosperous and a degraded one, $\mathcal{S} = \{\mathsf{g}, \mathsf{p}\}$.

```
env.Sset
```

```
['g', 'p']
```

In each state $s \in \mathcal{S}$, each agent $i \in \{1, \dots, N\}$ can choose from their action set between either cooperation or defection, $\mathcal{A}^i = \{\mathsf{c}, \mathsf{d}\}$.

```
env.Aset
```

```
[['c', 'd'], ['c', 'd']]
```

We denote the number of cooperating (defecting) agents by N_c ($N_\mathsf{d} = N - N_\mathsf{c}$).

A collapse from the prosperous state to the degraded state occurs with transition probability,

$$T(\mathsf{p}, a, \mathsf{g}) = \frac{N_\mathsf{d}}{N} q_c,$$

with $q_c \in [0, 1]$ being the collapse leverage parameter, indicating how much impact a defecting agent exerts on the environment. Thus, the environment remains within the prosperous state with probability, $T(\mathsf{p}, a, \mathsf{p}) = 1 - T(\mathsf{p}, a, \mathsf{g})$.

In the degraded state, we set the recovery to occur with probability,

$$T(\mathsf{g}, a, \mathsf{p}) = q_r,$$

independent of the agents' actions. The parameter q_r sets the recovery probability, and the probability that the environment remains degraded is, thus, $T(\mathsf{g}, a, \mathsf{g}) = 1 - T(\mathsf{g}, a, \mathsf{p})$.

```
env.T.round(4)
```

```
array([[[[0.99, 0.01],
         [0.99, 0.01]],

        [[0.99, 0.01],
         [0.99, 0.01]]],


       [[[0.  , 1.  ],
         [0.1 , 0.9 ]],

        [[0.1 , 0.9 ],
         [0.2 , 0.8 ]]]])
```

Rewards in the prosperous state follow the standard public good game,

$$R^i(\mathsf{p}, a^i a^{-i}, \mathsf{p}) = \begin{cases} fc\frac{N_c}{N} - c & \text{if } a^i = \mathsf{c} \\ fc\frac{N_c}{N} & \text{if } a^i = \mathsf{d} \end{cases}$$

where c denotes the cost of cooperation and f, the cooperation synergy factor.

```
env.R[0, 1, :, :, 1]
```

```
array([[ 1., -2.],
       [ 3.,  0.]])
```

```
env.R[1, 1, :, :, 1]
```

```
array([[ 1.,  3.],
       [-2.,  0.]])
```

However, when a state transition involves the degraded state, g, the agents receive an environmental collapse impact, $m < 0$,

$$R^i(\mathsf{p}, a, \mathsf{g}) = R^i(\mathsf{g}, a, \mathsf{p}) = R^i(\mathsf{g}, a, \mathsf{g}) = m, \quad \text{for all } a, i.$$

For illustration purposes, we set the model's parameters as $N = 2, f = 1.2, c = 5, m = -5, q_c = 0.2$, and $qr = 0.01$:

```
env = EPG(N=2, f=1.2, c=5, m=-5, qc=0.2, qr=0.01)
```

3.3 Reinforcement learning

Learning helps agents adjust their behavior to changes in their environment, both from other agents and external factors. This is essential when the future is unpredictable, unknown, and complex, and thus, detailed pre-planning is doomed to failure.

In particular, reinforcement learning is a trial-and-error method of mapping situations to actions to maximize a numerical reward signal (Sutton & Barto, 2018). When rewards are a delayed consequence of current actions, so-called temporal-difference or reward-prediction learning has been particularly influential (Sutton, 1988). This type of learning summarizes the difference between value estimates from past and present experiences into a reward-prediction error, which is then used to adapt the current behavior to gain more rewards over time. There also exist remarkable similarities between computational reinforcement learning and the results of neuroscientific experiments (Dayan & Niv, 2008). Dopamine conveys reward-prediction errors to brain structures where learning and decision-making occur (Schultz et al., 1997). This dopamine reward-prediction error signal constitutes a potential neuronal substrate for the essential economic decision quantity of *utility* (Schultz et al., 2017).

In the following, we present the essential elements of the reinforcement learning update.

3.3.1 Gain

We assume that at each time step t, each agent i strives to maximize its exponentially discounted sum of future rewards,

$$G_t^i = \mathsf{N}^i \sum_{k=0}^{\infty} (\gamma^i)^k r_{t+k}^i, \tag{3.1}$$

where $r^i(t+k)$ is the reward agent i receives at time step $t+k$, and $\gamma^i \in [0,1)$ is the discount factor of agent i. The *discount factor* regulates how much an agent cares for future rewards, where γ^i close to 1 means that it cares for the future almost as much for the present and γ^i close to 0 means that it cares almost only for immediate rewards. N^i denotes a normalization constant. It is either 1, or $(1-\gamma^i)$. While machine learning researchers often use $\mathsf{N}^i = 1$, the pre-factor $N^i = (1-\gamma^i)$ has the advantage of normalizing the gains, $G^i(t)$, to be on the same numerical scale as the rewards.

3.3.2 Value functions

Given a joint strategy X, we define the *state values*, $V_X^i(s)$, as the expected gain, $G^i(t)$, when starting in state s and then following the joint strategy, X,

$$V_X^i(s) = \mathbb{E}_X[G_t^i | s_t = s]. \tag{3.2}$$

Analogously, we define the *state-action values*, $Q_X^i(s,a)$, as the expected gain, $G^i(t)$, when starting in state s, executing action a, and then following the joint strategy, X,

$$Q_X^i(s,a) = \mathbb{E}_X[G_t^i | s_t = s, a_t^i = a]. \tag{3.3}$$

From Equation 3.1 and Equation 3.2, we can obtain the famous Bellman equation as follows, denoting the next state as s',

$$V^i_X(s) = \mathbb{E}_X[G^i_t | s_t = s] \tag{3.4}$$
$$= \mathbb{E}_X\Big[\mathsf{N}^i \sum_{k=0}^{\infty} (\gamma^i)^k r^i_{t+k} | s_t = s\Big] \tag{3.5}$$
$$= \mathbb{E}_X\Big[\mathsf{N}^i r^i_t + \mathsf{N}^i \gamma^i \sum_{k=0}^{\infty} (\gamma^i)^k r^i_{t+1+k} | s_t = s\Big] \tag{3.6}$$
$$= \mathbb{E}_X\Big[\mathsf{N}^i r^i_t + \gamma^i V^i_X(s') | s_t = s\Big] \tag{3.7}$$
$$= \mathbb{E}_X\Big[\mathsf{N}^i R^i(s, a, s') + \gamma^i V^i_X(s') | s_t = s\Big]. \tag{3.8}$$

Analogously, we can write for the state-action values,

$$Q^i_X(s, a) = \mathbb{E}_X\Big[\mathsf{N}^i R^i(s, a, s') + \gamma^i V^i_X(s') | s_t = s, a^i_t = a\Big]. \tag{3.9}$$

Thus, the value function can be expressed via a recursive relationship. The value of a state equals the discounted value of the next state ($\gamma^i V^i_X(s')$) plus the reward the agent receives along the way, properly normalized ($N^i R^i(s, a, s')$). This recursion will come in useful for learning (see Section 3.3.4).

3.3.3 Strategy function

In general, reinforcement learning agents do not know the true state and state-action values, $V^i_X(s)$, and $Q^i_X(s, a)$. Instead, they hold variable beliefs about the quality of each available action in each state $Q^i_t(s, a)$. The higher an agent believes an action brings value, the more likely it will choose it. We parameterize the agents' behavior according to the soft-max strategy function,

$$X^i_t(s, a) = \frac{e^{\beta^i Q^i_t(s,a)}}{\sum_b e^{\beta^i Q^i_t(s,b)}}, \tag{3.10}$$

where the *intensity-of-choice* parameters, $\beta^i \in \mathbb{R}^+$, regulate the exploration-exploitation trade-off. For high β^i, agents exploit their learned knowledge about the environment, leaning toward actions with high estimated state-action values. For low β^i, agents are more likely to deviate from these high-value actions to explore the environment further with the chance of finding actions that eventually lead to even higher values. This soft-max strategy function can be motivated by the maximum-entropy principle (Jaynes & Bretthorst, 2003), stating that the current strategy of an agent should follow a distribution that maximizes entropy subject to current beliefs about the qualities $Q^i_t(s, a)$ (Wolpert, 2006; Wolpert et al., 2012).

3.3.4 Learning

Learning means updating the quality estimates, $Q^i_t(s, a)$, with the current reward-prediciton error, $\delta^i_t(s, a)$, after selection action a_t in state s_t according to

$$Q^i_{t+1}(s_t, a_t) = Q^i_t(s_t, a_t) + \alpha^i \delta^i_t(s_t, a_t), \tag{3.11}$$

where $\alpha^i \in (0,1)$ is the learning rate of agent i, which regulates how much new information the agent uses for the update. The reward-prediction error, $\delta_t^i(s_t, a_t)$, equals the difference of the new quality estimate, $\mathsf{N}^i r_t^i + \gamma^i \mathcal{Q}_n^i(s_{t+1})$, and the current quality estimate, $\mathcal{Q}_c^i(s_t)$,

$$\delta_t^i(s_t, a_t) = \mathsf{N}^i r_t^i + \gamma^i \mathcal{Q}_n^i(s_{t+1}, a_{t+1}) - \mathcal{Q}_c^i(s_t, a_t), \tag{3.12}$$

where the $\mathcal{Q}_n^i$ represents the quality estimate of the *next* state and $\mathcal{Q}_c^i$ represents the quality estimate of the *current* state. Depending on how we choose, $\mathcal{Q}_n^i$, and $\mathcal{Q}_c^i$, we recover various well-known temporal-difference reinforcement learning update schemes (Barfuss et al., 2019).

Variants

For example, if $\mathcal{Q}_n^i = \mathcal{Q}_c^i = Q_t^i$, we obtain the so called SARSA update,

$$\delta_t^i(s_t, a_t) = \mathsf{N}^i r_t^i + \gamma^i Q_t^i(s_{t+1}, a_{t+1}) - Q_t^i(s_t, a_t).$$

If $\mathcal{Q}_n^i = \max_b Q_t^i(s_{t+1}, b)$, and $\mathcal{Q}_c^i = Q_t^i$, we obtain the famous Q-learning update,

$$\delta_t^i(s_t, a_t) = \mathsf{N}^i r_t^i + \gamma^i \max_b Q_t^i(s_{t+1}, b) - Q_t^i(s_t, a_t).$$

And if $\mathcal{Q}_n^i = \mathcal{Q}_c^i = V_t^i$ is a separate state-value estimate, we obtain an actor-critic update,

$$\delta_t^i(s_t, a_t) = \mathsf{N}^i r_t^i + \gamma^i V_t^i(s_{t+1}) - V_t^i(s_t).$$

3.4 Collective Reinforcement Learning Dynamics (CRLD)

3.4.1 Motivation

In Section 3.3, we saw how to derive temporal-difference reward-prediction reinforcement learning from first principles. Agents strive to improve their discounted sum of future rewards (Equation 3.1) while acting according to the maximum entropy principle (Equation 3.10). However, using these standard reinforcement algorithms directly for modeling comes also with some *challenges*:

- First of all, the learning is highly *stochastic*, since, in general, all agents strategies $X^i(s,a)$, and the environments transition function $T(s, a, s')$ are probability distributions.
- This stochasticity can make it sometimes *hard to explain*, why a phenomenon occurred in a simulation.
- Reinforcement learning is also very *sample-inefficient*, meaning it can take the agents a long time to learn something.
- Thus, learning simulations are *computationally intense*, since one requires many simulations to make sense of the stochasticity, of which each takes a long time to address the sample inefficiency.

How can we address these challenges? In Section 3.3.4, we saw that we could express different reward-prediction learning variants by formulating different reward-prediction errors, δ. The essential idea of the collective reinforcement learning dynamics approach is to replace the individual sample realizations of the reward-prediction error with its strategy average plus a small error term,

$$\delta \leftarrow \bar{\delta} + \epsilon.$$

Thus, collective reinforcement learning dynamics describe how agents with access to (a good approximation of) the strategy-average reward-prediction error would learn. There are at least three interpretations to motivate how the agents can obtain the strategy averages:

- The agents are batch learners. They store experiences (state observations, rewards, actions, next state observations) inside a memory batch and replay these experiences to make the learning more stable. In the limit of an infinite memory batch, the error term vanishes, $\epsilon \to 0$ (Barfuss, 2020).
- The agents learn on two different time scales. On one time scale, the agents interact with the environment, collecting experiences and integrating them to improve their quality estimates while keeping their strategies fixed. On the other time scale, they use the accumulated experiences to adapt their strategy. In the limit of a complete time scale separation, having infinite experiences between two strategy updates, the error term vanishes, $\epsilon \to 0$ (Barfuss, 2022).
- The agents have a model of how the environment works, including how the other agents behave currently, but not how the other agents learn. This model can be used to stabilize learning. In the limit of a perfect model (and sufficient cognitive resources), the error term vanishes, $\epsilon \to 0$.

In the following, we focus on the idealized case of a vanishing error term, $\epsilon \to 0$.

3.4.2 Derivation

We start by combining Equation 3.10 and Equation 3.11 to obtain the joint strategy update,

$$X^i_{t+1}(s,a) = \frac{X^i_t(s,a)\exp\left(\alpha^i\beta^i\bar{\delta}^i(s,a)\right)}{\sum_b X^i_t(s,b)\exp\left(\alpha^i\beta^i\bar{\delta}^i(s,b)\right)}, \tag{3.13}$$

where we have also replaced the sample reward-prediction error, $\delta^i_t(s,a)$, with its strategy average, $\bar{\delta}^i(s,a)$. Thus, in the remainder, we can focus on obtaining the strategy-average reward-prediction error, $\bar{\delta}^i(s,a) = \delta^i_{\mathbf{X}_t}(s,a)$. We equip a symbol with a straight bar on top to denote the averaging with the current joint policy $\mathbf{X}_t$. From Equation 3.12, we see that we need to construct the strategy-average reward, the strategy-average value of the next state, and the strategy-average value of the current state.

Equation 3.13 suggests summarizing the product of the learning rate α^i and the intensity-of-choice β^i into an effective learning rate η^i. If we restate the denominator by $\bar{\mathfrak{Z}}^i(s) = \sum_b X^i_t(s,b)\exp\left(\alpha^i\beta^i\bar{\delta}^i(s,b)\right)$, we recover exactly the form used in the main text,

$$X^i_{t+1}(s,a) = \frac{1}{\bar{\mathfrak{Z}}^i(s)} X^i_t(s,a)\exp\left(\eta^i \cdot \bar{\delta}^i(s,a)\right).$$

Rewards

The strategy-average version of the current reward is obtained by considering each agent i taking action a in state s when all other agents j act according to their strategy $X^j(s,a^j)$, causing the environment to transition to the next state s' with probability $T(s,a,s')$, during which agent i receives reward $R^i(s,a,s')$. Mathematically, we write,

$$\bar{R}^i(s,a) = \sum_{a^j}\sum_{s'}\prod_{j\neq i} X^j(s,a^j)T(s,a,s')R^i(s,\mathbf{a},s').$$

Next values

The strategy average of the following state value is likewise computed by averaging over all actions of the other agents and following states.

We start with the simplest learning variant, *actor-critic learning*. For each agent i, state s, and action a, all other agents $j \neq i$ choose their action a^j with probability $X^j(s, a^j)$. Consequently, the environment transitions to the next state s' with probability $T(s, a, s')$. At s', the agent estimates the quality of the next state to be of $\bar{V}^i(s')$. Mathematically, we write,

$$^{n}\bar{Q}^i(s,a) = \sum_{a^j} \sum_{s'} \prod_{j \neq i} X^j(s, a^j) T(s, a, s') \bar{V}^i(s').$$

We obtain the strategy-average value estimate of the following state precisely as the state values of the following state, $\bar{V}^i(s') = V^i_X(s')$, as defined in Equation 3.2. We compute them by writing the Bellman equation,

$$\bar{V}^i(s) = \mathsf{N}^i \bar{R}^i(s) + \gamma^i \bar{T}(s, s') \bar{V}^i(s'),$$

in matrix form,

$$\bar{V}^i = \mathsf{N}^i \bar{R}^i + \gamma^i \bar{T} \bar{V}^i,$$

which allows us to bring all state value variables on one site through a matrix inversion,

$$\bar{V}^i = N^i \left(\mathbb{1}_Z - \gamma^i \bar{T} \right)^{-1} \bar{R}^i.$$

Here, $\bar{R}^i(s)$ is the strategy-average reward value agent i receives in state s. They are computed by averaging over all agents' strategies, $X^j(s, a^j)$, and the state transition $T(s, a, s')$,

$$\bar{R}^i(s) = \sum_{a^j} \sum_{s'} \prod_{j} X^j(s, a^j) T(s, a, s') R^i(s, a, s').$$

And $\bar{T}(s, s')$ are the strategy-average transition probabilities. They are computed by averaging over all agents' strategies, $X^j(s, a^j)$,

$$\bar{T}(s, s') = \sum_{a^j} \prod_{j} X^j(s, a^j) T(s, a, s').$$

Last, $\mathbb{1}_Z$, is the Z-by-Z identity matrix.

For *SARSA learning*, the strategy average of the following state value reads,

$$^{n}\bar{Q}^i(s,a) = \sum_{a^j} \sum_{s'} \prod_{j \neq i} X^j(s, a^j) T(s, a, s') \sum_{a^i} X^i(s', a^i) \bar{Q}^i(s', a^i),$$

where we replace $Q^i_t(s_{t+1}, a_{t+1})$ by the strategy-average next-state next-action value $\sum_{a^i} X^i(s', a^i) \bar{Q}^i(s', a^i)$.

Here, the strategy-average state-action values, $\bar{Q}^i(s,a) = Q^i_X(s,a)$, are exaclty the state-action values defined in Equation 3.3. We compute them exactly as Equation 3.3 prescribes,

$$\bar{Q}^i(s,a) = \mathsf{N}^i \bar{R}^i(s,a) + \gamma^i \sum_{s'} \bar{T}^i(s, a, s') \bar{V}^i(s'),$$

where $\bar{T}^i(s,a,s')$ is the strategy-average transition model from the perspective of agent i. It can be computed by averaging out all other agents' strategies from the transition tensor,

$$\bar{T}^i(s,a,s') = \sum_{a^j} \prod_{j \neq i} X^j(s,a^j) T(s,\mathbf{a},s').$$

However, it is easy to show that $\sum_{a^i} X^i(s',a^i)\bar{Q}^i(s',a^i) = \bar{V}^i(s')$, and thus, the strategy-average next-state values of SARSA and actor-critic learning are indeed identical.

Current values

The strategy-average of the current state value in the reward-prediction error of *actor-critic learning*, $\bar{V}^i(s)$, is - for each agent i and state s - a constant in actions. Thus, they do not affect the joint strategy update (Equation 3.13).

The state-action value of the current state, $Q^i_t(s_t,a_t)$, in *SARSA learning* becomes, $\frac{1}{\beta^i}\ln X^i(s,a)$, in the strategy-average reward-prediction error and can be seen as a regularization term. We can derive it by inverting Equation 3.10,

$$Q^i_t(s,a) = \frac{1}{\beta^i}\ln X^i_t(s,a) + \frac{1}{\beta^i}\ln\left(\sum_b e^{\beta^i Q^i_t(s,b)}\right),$$

and realizing that the dynamics induced by Equation 3.13 are invariant under additive transformations, which are constant in actions.

Reward-prediction error

Together, the strategy-average reward-prediction error for *actor-critic learning* reads,

$$\bar{\delta}^i(s,a) = \mathsf{N}^i\bar{R}^i(s,a) + \gamma^i \cdot {}^n\bar{Q}^i(s,a) = \bar{Q}^i(s,a),$$

and the strategy-average *actor-critic learning* dynamics, thus,

$$X^i_{t+1}(s,a) = \frac{X^i_t(s,a)\exp\left(\alpha^i\beta^i\bar{Q}^i(s,a)\right)}{\sum_b X^i_t(s,b)\exp\left(\alpha^i\beta^i\bar{Q}^i(s,b)\right)}.$$

With $\alpha^i\beta^i\bar{Q}^i(s,a)$ being the *fitness* of agent i's action a in state s, these dynamics are exactly equivalent to the alternative replicator dynamics in discrete time (Hofbauer & Sigmund, 2003).

For *SARSA learning*, the strategy-average reward-prediction error reads,

$$\bar{\delta}^i(s,a) = \mathsf{N}^i\bar{R}^i(s,a) + \gamma^i \cdot {}^n\bar{Q}^i(s,a) - \frac{1}{\beta^i}\ln X^i(s,a) = \bar{Q}^i(s,a) - \frac{1}{\beta^i}\ln X^i(s,a),$$

and the strategy-average *SARSA learning* dynamics, thus,

$$X^i_{t+1}(s,a) = \frac{X^i_t(s,a)\exp\left(\alpha^i(\beta^i\bar{Q}^i(s,a) - \ln X^i(s,a))\right)}{\sum_b X^i_t(s,b)\exp\left(\alpha^i(\beta^i\bar{Q}^i(s,b) - \ln X^i(s,b))\right)}.$$

4 Multi-stability

In this section, we illustrate complex phenomena around multi-stability in the phase space of CRLD.

First, we import everything we need:

```
import numpy as np
import matplotlib.pyplot as plt
from matplotlib.lines import Line2D

from pyCRLD.Environments.EcologicalPublicGood import EcologicalPublicGood as EPG
from pyCRLD.Agents.StrategyActorCritic import stratAC
from pyCRLD.Utils import FlowPlot as fp
```

4.1 Phase space plot

We start by plotting the flow of CRLD in the strategy phase space projection of the prosperous state. For that, we define a function, to help us compile as initial strategies.

```
def compile_strategy(p0c:float,  # cooperation probability of agent zero
                     p1c:float):  # cooperation probability of agent one
    Pi = np.array([0.95, p0c])  # coop. prob. in degraded state set to 0.95
    Pj = np.array([0.95, p1c])
    xi = np.array([Pi, 1-Pi]).T
    xj = np.array([Pj, 1-Pj]).T
    return np.array([xi, xj])
```

For example,

```
compile_strategy(0.2, 0.95)
```

```
array([[[0.95, 0.05],
        [0.2 , 0.8 ]],

       [[0.95, 0.05],
        [0.95, 0.05]]])
```

The arrows indicate the strategy-average reward-prediction errors. Their colors additionally indicate their length.

```python
# Inititalize the ecological public good environment
env = EPG(N=2, f=1.2, c=5, m=-5, qc=0.2, qr=0.01, degraded_choice=False)

# Create multi-agent environment interface
MAEi = stratAC(env=env, learning_rates=0.1, discount_factors=0.75)

# Strategy flow plot
# -------------------
x = ([0], [1], [0])  # which (agent, observation, action) to plot on x axis
y = ([1], [1], [0])  # which (agent, observation, action) to plot on y axis
eps=10e-3; action_probability_points = np.linspace(0+eps, 1.0-eps, 9)
ax = fp.plot_strategy_flow(MAEi, x, y, action_probability_points, NrRandom=64)

# Trajectories
# -------------------
xtrajs = []  # storing strategy trajectories
fprs = []    # and whether a fixed point is reached
for pc in [0.15, 0.175, 0.2]:  # cooperation probability of agent 2
    X = compile_strategy(pc, 0.95)
    xtraj, fixedpointreached = MAEi.trajectory(X, Tmax=2000, tolerance=10**-5)
    xtrajs.append(xtraj); fprs.append(fixedpointreached)
    print("Trajectory length:",len(xtraj))

# Add trajectories to flow plot
fp.plot_trajectories(xtrajs, x=x, y=y, fprs=fprs,
                     cols=['red','blue','blue'], lws=[2], msss=[2],
                     lss=['-'], alphas=[0.75],
                     axes=ax)

# Add separatrix
o = [0.619, 0.6191]; o1 = compile_strategy(*o); o2 = compile_strategy(*o[::-1])
sep1=[]; sep2=[]
for _ in range(1000): o1, _ = MAEi.reverse_step(o1); sep1.append(o1)
for _ in range(1000): o2, _ = MAEi.reverse_step(o2); sep2.append(o2)
fp.plot_trajectories([sep1, sep2], x=x, y=y, cols=['purple'], lws=[1],
                     lss=['--'], alphas=[0.95], plot_startmarker=False, axes=ax)

# Add saddle node
# by reversing the dynamics from two agents with identical strategies
o = [0.5, 0.5]; o = compile_strategy(*o)
for _ in range(1000): o, _ = MAEi.reverse_step(o)
ax[0].scatter(*o[:,1,0], c='purple', marker='P', s=50)

# Make labels nice
ax[0].set_ylabel(f"$X^2(s=Prosp.,a=Coop.)$")
ax[0].set_xlabel(f"$X^1(s=Prosp.,a=Coop.)$")

# # Save plot
plt.gcf().set_facecolor('white') # for dark mode on web
plt.tight_layout()
plt.savefig('_figs/fig_01PhaseSpace.png', dpi=150)
```

```
Trajectory length: 296
Trajectory length: 298
Trajectory length: 253
```

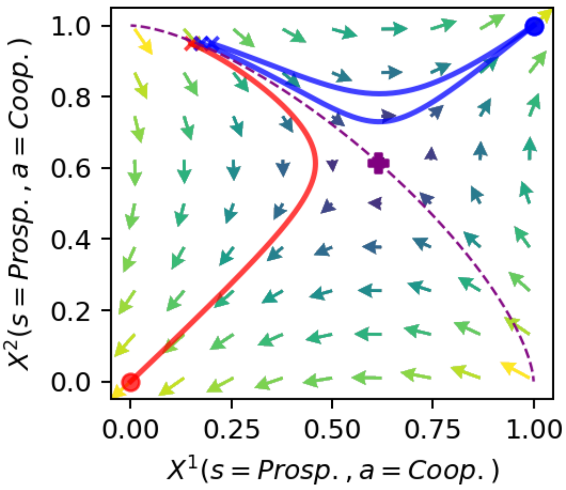

Figure 4.1: Phase space projection of the prosperous state of the ecological public goods environment

4.2 Sample trajectories

Next, we create a more fine-grained bundle of learning trajectories.

```
# Cooperation probability of agent 2
pcs = np.concatenate((np.linspace(0.05, 0.95, 51),
                       np.linspace(0.15, 0.18, 101),
                       np.linspace(0.1646, 0.1649, 101),
                       np.linspace(0.16475, 0.164765, 51)))
pcs = np.sort(np.unique(pcs))

xtrajs = []  # storing strategy trajectories
fprs = []    # and whether a fixed point is reached
for pc in pcs:
    # Compile initial joint strategy
    Pi = np.array([0.95, pc])
    Pj = np.array([0.95, 0.95])
    xi = np.array([Pi, 1-Pi]).T
    xj = np.array([Pj, 1-Pj]).T
    X = np.array([xi, xj])

    # Compute trajectory
    xtraj, fixedpointreached = MAEi.trajectory(X, Tmax=2000, tolerance=10**-5)
    xtrajs.append(xtraj)
    fprs.append(fixedpointreached)
```

We obtain the critical point in this bundle of learning trajectories where the two agents switch or tip from complete defection to complete cooperation.

```
# assuming, that all trajectories convergend
assert np.all(fprs)

# obtaining the cooperation probability at convergences
converged_pcs = [xtraj[-1][:, 1, 0] for xtraj in xtrajs]

# showing the biomodal distribution of full defection and full cooperation
np.histogram(np.array(converged_pcs).mean(-1), range=(0,1))[0]
```

```
array([138,   0,   0,   0,   0,   0,   0,   0,   0, 162])
```

Thus, the critical point lies at the index

```
cp = np.histogram(np.array(converged_pcs).mean(-1), range=(0,1))[0][0]
cp
```

```
138
```

and has an approximate value between

```
print(pcs[cp-1], 'and', pcs[cp], '.')
```

```
0.1647584 and 0.1647587 .
```

4.3 Critical slowing down

We use this more fine-grained bundle of learning trajectories to visualize the phenomenon of a *critical slowing down* by plotting the time steps required to reach convergence.

```
# Create the canves
fsf = 0.7 # figure size factor
plt.figure(figsize=(fsf*4, fsf*2.5))

# Plot the time steps required to convergence, i.e. the trajectory lengths
plt.plot(pcs[:cp], [len(xtraj) for xtraj in xtrajs[:cp]],
         '-', color='red', lw=2, alpha=0.8)  # defectors in red
plt.plot(pcs[cp:], [len(xtraj) for xtraj in xtrajs[cp:]],
         '-', color='blue', lw=2, alpha=0.6) # cooperators in blue

# Make labels and axis nice
plt.xlabel(f"$X^1(s=Prosp.,a=Coop.)$")
plt.ylabel('Timesteps to\nconvergence')
plt.xlim(0,1)
plt.ylim(0, 800)
plt.gca().spines.right.set_visible(False)
plt.gca().spines.top.set_visible(False)
```

```
# Save plot
plt.gcf().set_facecolor('white') # for dark mode on web
plt.subplots_adjust(top=0.95, bottom=0.3, left=0.28, right=0.94)
plt.savefig('_figs/fig_01SlowingDown.png', dpi=150)
```

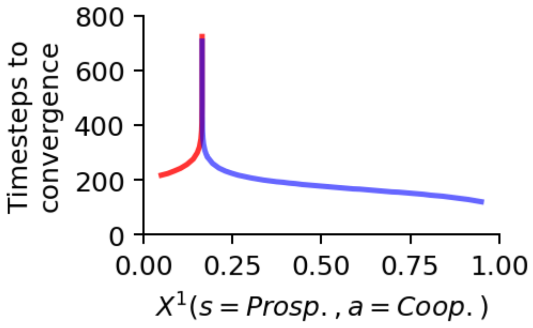

Figure 4.2: Time steps required to convergence show a critical slowing down around the tipping point.

At the critical point $X_0^1(s = \text{Prosp.}, a = \text{Coop.}) \approx 0.16475855$, the collective learning takes about an order of magnitude longer to converge than close to full cooperation $X_0^1(s = \text{Prosp.}, a = \text{Coop.}) \approx 1.0$, and about four times as much than close to full defection $X_0^1(s = \text{Prosp.}, a = \text{Coop.}) \approx 0.0$.

4.4 Detailed phase space

We plot a detailed phase space where we zoom in on the area around the critical saddle point on the separatrix.

```
# Create the canves
fsf =  0.65  # figure size factor
_, ax = plt.subplots(1,1, figsize=(fsf*4, fsf*3.5))

# Plot the reward-prediction error flow
action_probability_points = np.linspace(0.612, 0.619, 17)
ax = fp.plot_strategy_flow(MAEi, x, y, action_probability_points, NrRandom=64,
                           axes=[ax])

# Plot the defecting learning trajectories in red
fp.plot_trajectories(xtrajs[:cp], x=x, y=y, fprs=fprs, axes=ax, cols=['red'],
                     lws=[2], msss=[2], mss=['.'], lss=['-'], alphas=[0.15])

# Plot the cooperating learning trajectories in blue
fp.plot_trajectories(xtrajs[cp:], x=x, y=y, fprs=fprs, axes=ax, cols=['blue'],
                     lws=[2], msss=[2], mss=['.'], lss=['-'], alphas=[0.15])

# Make labels and axis nice
ax[0].set_ylabel(f"$X^2(s=Prosp.,a=Coop.)$")
ax[0].set_xlabel(f"$X^1(s=Prosp.,a=Coop.)$")

ax[0].set_ylim(0.613, 0.619)
```

```
ax[0].set_xlim(0.6125, 0.6155)

# Save plot
plt.gcf().set_facecolor('white') # for dark mode on web
plt.tight_layout()
plt.savefig('_figs/fig_01PhaseSpaceDetail.png', dpi=150)
```

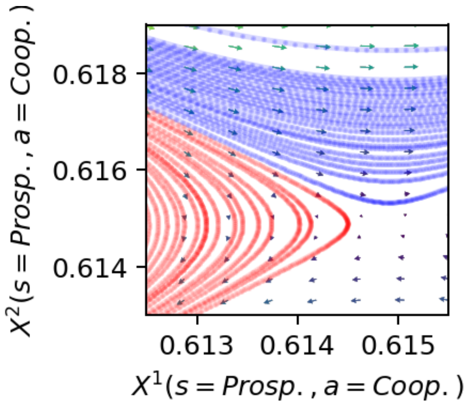

Figure 4.3: Strategy phase space at the critical bifurcation point.

4.5 Time scale separation

Last, we visulize the emergent time scale separation at the critical point by plotting the level of cooperation over time for the two initial strategies around the critical point.

```
# Create the canves
fsf = 0.5  # figure size factor
plt.figure(figsize=(fsf*6, fsf*4))

# Plot the defecting learners in red
#   agent 1 with dots
plt.plot(xtrajs[cp-1][:, 0, 1, 0], color='red', lw=5, ls=':')
#   agent 2 with dashes
plt.plot(xtrajs[cp-1][:, 1, 1, 0], color='red', lw=4, ls="--", alpha=0.4)

# Plot the cooperating learners in blue
#   agent 1 with dots
plt.plot(xtrajs[cp][:, 0, 1, 0], color='blue', lw=3, ls=':')
#   agent 2 with dashes
plt.plot(xtrajs[cp][:, 1, 1, 0], color='blue', lw=2, ls="--", alpha=0.4)

# Create a nice legend
custom_lines = [Line2D([0], [0], color='black', ls=':', lw=2),
                Line2D([0], [0], color='gray', ls='--', lw=2)]
plt.legend(custom_lines, ['Agent 1', 'Agent 2'], ncol=1)

# Make labels and axis nice
plt.gca().spines.right.set_visible(False)
```

```
plt.gca().spines.top.set_visible(False)
plt.xlabel("Timesteps")
plt.ylabel("Cooperation")

# Save plot
plt.gcf().set_facecolor('white') # for dark mode on web
plt.subplots_adjust(top=0.98, bottom=0.22, left=0.22, right=0.98)
plt.savefig('_figs/fig_01PhaseSpaceTrajectory.png', dpi=150)
```

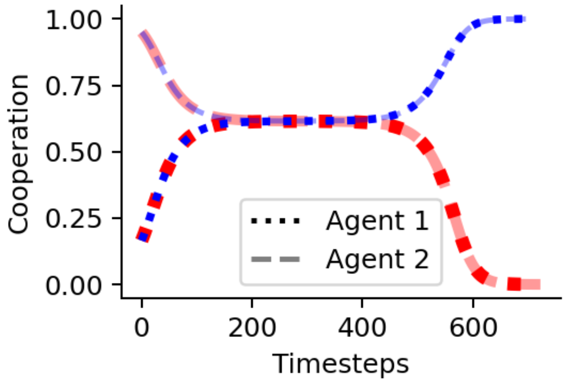

Figure 4.4: Emergent time scale seperation at the critical point.

5 Abrupt transitions

In this section, we illustrate complex phenomena around abrupt transitions in the parameter space of CRLD. In this example, we focus on the discount factor, which indicates how much the agents care for future rewards. Abrupt transitions go by many names, such as critical transitions, regime shifts, bifurcations, or tipping elements, to name some of them.

First, we import everything we need:

```
import numpy as np
import matplotlib.pyplot as plt

from pyCRLD.Environments.EcologicalPublicGood import EcologicalPublicGood as EPG
from pyCRLD.Agents.StrategyActorCritic import stratAC

import _code.SimulationScripts as sim
```

5.1 Compute data

We start by computing the CRLD trajectories from 250 random initial strategies along a varying discount factor.

```
# Set data trajectory for storing results, e.g.,
ddir = '/Users/wolf/Downloads/CoCoIn_data'

# Initialize first environment and multi-agent environment interface (MAEi)
env = EPG(N=2, f=1.2, c=5, m=-5, qc=0.2, qr=0.01, degraded_choice=False)
MAEi = stratAC(env=env, learning_rates=0.1, discount_factors=0.99,
 ↪  use_prefactor=True)

# Create random initial strategies from simulation scripts 'sim'
Xs = sim.initial_strategies(MAEi, 250)

# Create discount factors to loop through
discountfacts = np.sort(np.unique(list(np.linspace(0.1, 1.0, 10)[:-1].round(2))
                        + list(np.arange(0.5, 1.0, 0.05).round(2))
                        + list(np.arange(0.65, 0.9, 0.0125).round(4))
                        + [0.01, 0.99]))
print("Discount factors:")
print(discountfacts)
```

```
Discount factors:
[0.01   0.1    0.2    0.3    0.4    0.5    0.55   0.6    0.65   0.6625
 0.675  0.6875 0.7    0.7125 0.725  0.7375 0.75   0.7625 0.775  0.7875
```

```
 0.8    0.8125 0.825  0.8375 0.85   0.8625 0.875  0.8875 0.9    0.95
 0.99  ]
```

```
# compute or load the data from disk (if they exist)
convtimes = []; rewss = []; coops = []
for dcf in discountfacts:
    print(f" = = = = {dcf} = = = =")
    MAEi = stratAC(env=env, learning_rates=0.1, discount_factors=dcf,
                   use_prefactor=False)

    trjs, fprs = sim.obtain_trajectories(MAEi, Xs, 25000, ddir=ddir)

    # convergence times
    convtimes.append([len(trj) for trj in trjs])

    # final rewards
    rewss.append(sim.final_rewards(MAEi, trjs))

    # cooperative acts
    coops.append([trj[-1].astype(float)[:,1,0] for trj in trjs])
```

5.2 Plotting function

Next, we create a function to plot the data along the varying parameter.

```
def plot_valuehistograms_vs_parameters(parameters, values, bins, rnge,
                                       marker='.', alpha=1.0, color='black',
                                       cmap='viridis', ax=None):
    """
    Plot a histogram for each parameter next to each other.

    Parameters
    ----------
    parameters: iterable
        of float-valued parameters
    values: iterable
        of iterable of values for each parameter
    bins: int
        The number of bins for the histograms
    rnge: tupe
        Range of the histogram as (min, max)
    """
    # Figure
    if ax is None:
        _, ax = plt.subplots()

    # Create iterable of histograms for values
    valhist=[]
    for conv in values:
        h = np.histogram(conv, bins=bins, range=rnge)[0]
```

```
        valhist.append(h)

    # Adjust spacing
    params = np.array(parameters)
    delta = params[1:] - params[:-1]
    paramedges = np.concatenate(([parameters[0]-0.5*delta[0]],
                                 parameters[:-1] + 0.5*delta,
                                 [parameters[-1]+0.5*delta[-1]]))
    valedges = np.linspace(rnge[0], rnge[1], bins+1)

    # Plot histograms with colormap
    X, Y = np.meshgrid(paramedges, valedges)
    ax.pcolormesh(X, Y, np.array(valhist).T, cmap=cmap, alpha=alpha*0.75)

    # Plot median, quantiles and mean
    quartile1, medians, quartile3 = np.percentile(values, [25, 50, 75], axis=1)
    ax.fill_between(params, quartile1, quartile3, color=color, alpha=alpha*0.2)
    ax.plot(params, medians, marker=marker, markersize=4, linestyle='-',
            color=color, alpha=0.5*alpha)
    ax.plot(params, np.mean(values, axis=1), marker=marker, linestyle='',
            color=color, alpha=alpha)

    # Adjust the visible y range
    ax.set_ylim(rnge[0], rnge[1])
```

5.3 Abrupt transition

We use the created plotting function (Section 5.2) to visualize the phenomenon of an abrupt transition from complete defection to complete cooperation.

We show the abrupt transition in the level of cooperation at convergence.

```
# Create the canves
fsf = 0.7  # figure size factor
fig, ax = plt.subplots(figsize=(fsf*6, fsf*3))

# Plot the cooperation probabilities versus the discount factors
plot_valuehistograms_vs_parameters(parameters=discountfacts,
                                   values=np.array(coops).mean(-1), ax=ax,
                                   bins=21, rnge=(-0.1, 1.1), cmap='Blues')

# Make labels and axis nice
plt.ylabel('Cooperation')
plt.xlabel('Discount factor')

# Save plot
plt.subplots_adjust(left=0.15, right=0.98, top=0.98, bottom=0.2)
```

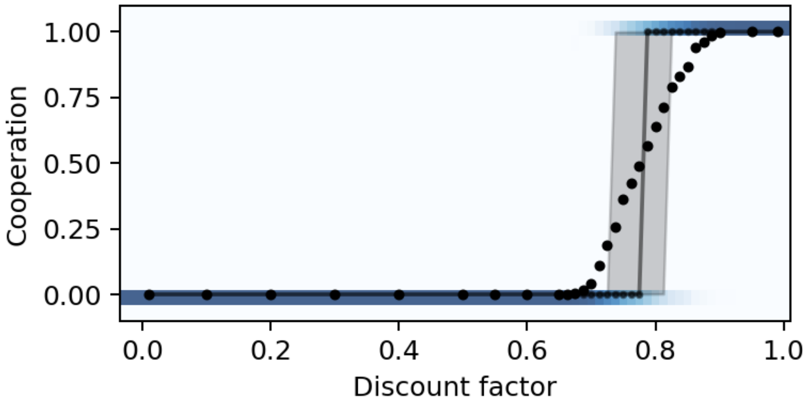

Figure 5.1: Abrupt transition in final cooperation likelihood

We also show the abrupt transition in the level of final rewards obtained by the agents.

```
# Create the canves
fsf = 0.7  # figure size factor
fig, ax = plt.subplots(figsize=(fsf*6, fsf*3))

# Plot the reward levels versus the discount factors
plot_valuehistograms_vs_parameters(parameters=discountfacts,
                                   values=np.array(rewss).mean(-1), ax=ax,
                                   bins=21, rnge=(-5.25, 1.25), cmap='Reds')

# Make labels and axis nice
plt.ylabel('Reward')
plt.xlabel('Discount factor')
plt.subplots_adjust(left=0.15, right=0.98, top=0.98, bottom=0.2)
```

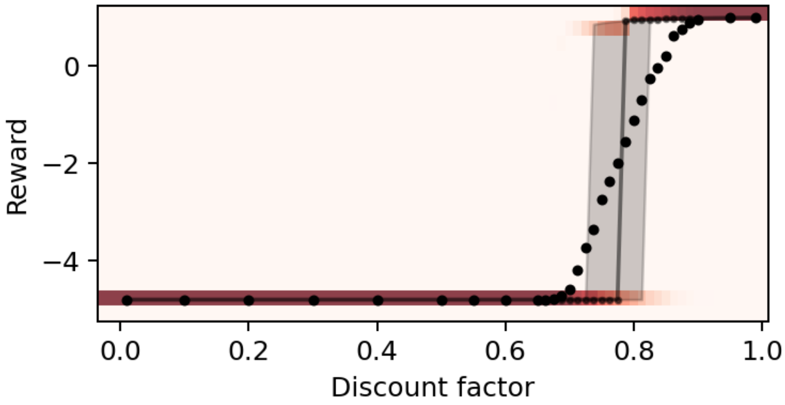

Figure 5.2: Abrupt transition in final rewards

Since the transition from complete defection to complete cooperation and from low reward and high reward appear similar, we can also try to plot them together into one plot, with cooperation on the left y-axis and the reward on the right y-axis.

```
# Create the canves
fsf = 0.7  # figure size factor
fig, ax1 = plt.subplots(figsize=(fsf*6, fsf*2.8))
ax2 = ax1.twinx()  # instantiate a second axes that shares the same x-axis

# Plot the cooperation probabilities versus the discount factors
plot_valuehistograms_vs_parameters(parameters=discountfacts,
                                   values=np.array(coops).mean(-1), ax=ax1,
                                   bins=21, rnge=(-0.1, 1.1), cmap='Blues',
                                   marker='x', color='blue')

# Plot the reward levels versus the discount factors
plot_valuehistograms_vs_parameters(parameters=discountfacts,
                                   values=np.array(rewss).mean(-1), ax=ax2,
                                   bins=21, rnge=(-5.35, 1.55), cmap='Reds',
                                   marker='.', alpha=0.5, color='red')

# Make labels and axis nice
ax1.set_xlabel('Discount factor')
ax1.set_ylabel('Cooperation (X)', color='Blue')
ax1.tick_params(axis='y', labelcolor='Blue')
ax2.set_ylabel('Reward ( )', color='Red')
ax2.tick_params(axis='y', labelcolor='Red')
ax2.set_yticks([-5, 0, 1]);
plt.subplots_adjust(left=0.15, right=0.88, top=0.96, bottom=0.22)
plt.savefig('_figs/fig_02AbruptTransitionCooperationReward.png', dpi=150)
```

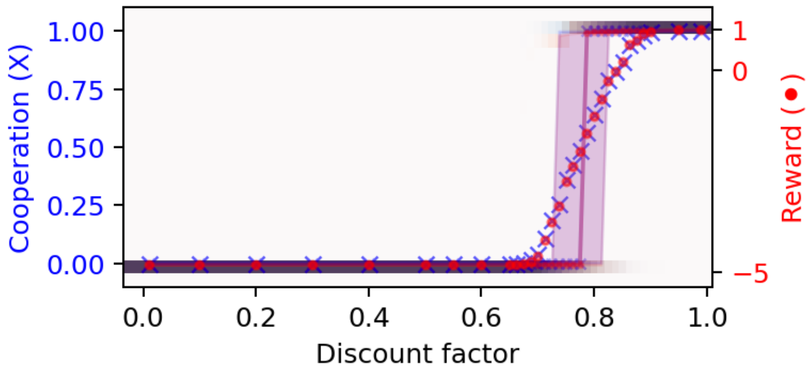

Figure 5.3: Abrupt transition in final cooperation likelihood and in final rewards

5.4 Critical slowing down

We use the created plotting function (Section 5.2) to visualize the phenomenon of a critical slowing down of the learning speed around the tipping point.

```
# Create the canves
fsf = 0.7 # figure size factor
fig, ax = plt.subplots(figsize=(fsf*6, fsf*3.5))
```

```
# Plot the convergence times versus the discount factors
plot_valuehistograms_vs_parameters(parameters=discountfacts, values=convtimes,
                                   bins=21, rnge=(0, 150), cmap='Greys', ax=ax)

# Make labels and axis nice
plt.ylabel('Timesteps to convergence')
plt.xlabel('Discount factor')

# Save plot
plt.subplots_adjust(left=0.15, right=0.98, top=0.96, bottom=0.18)
plt.savefig('_figs/fig_02AbruptTransitionSpeed.png', dpi=150)
```

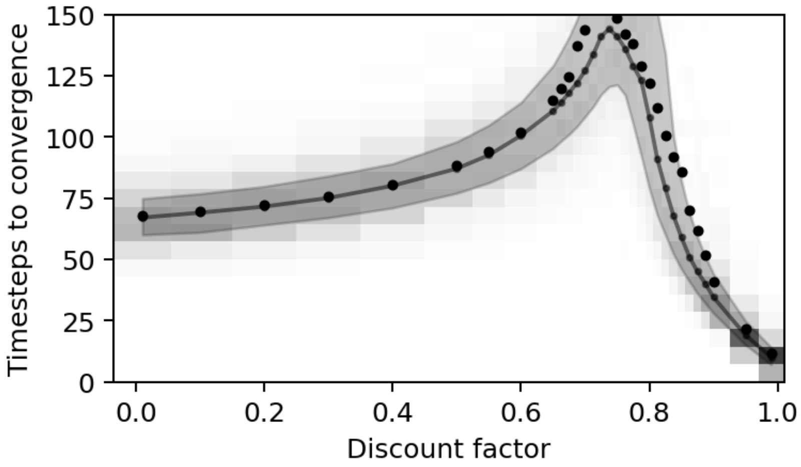

Figure 5.4: Critical slowing down in parameter space

6 Hysteresis

In this section, we illustrate the complex phenomenon of hysteresis in CRLD. Hysteresis means that the system's state depends on the history of external parameter changes. Here, we show that hysteresis exists by varying the discount factor, which indicates how much the agents care for future rewards. We let the discount factor increase and decrease again while the CRLD keeps running.

First, we import everything we need:

```
import numpy as np
import matplotlib.pyplot as plt

from pyCRLD.Environments.EcologicalPublicGood import EcologicalPublicGood as EPG
from pyCRLD.Agents.StrategySARSA import stratSARSA as stratS
```

In contrast to the previous examples, where we used `stratAC`, i.e., actor-critic learning agents in strategy space, we use `stratSARSA` agents, as seen above in the imports. The SARSA agents differ from the actor-critic learners in their exploration terms. The SARSA agents keep a constant exploration term, which prevents them from converging too close to the edges of the strategy phase space. They are constantly exploring to some extent. Keeping a small distance to the edges of the strategy phase space is required for hysteresis. When the external parameter changes while CRLD keeps running, the agents need to be able to change their current equilibrium. Otherwise, no change of equilibrium is observable.

To be able to change their current equilibrium requires the agents to keep a small distance from the strategy phase space edges, as one can also see in the learning update equation,

$$X^i_{t+1}(s,a) = \frac{1}{\mathfrak{Z}^i(s)} X^i_t(s,a) \exp\left(\eta^i \cdot \bar{\delta}^i(s,a)\right).$$

If $X^i_t(s,a)$ is too close to zero or one, no update can happen, regardless of the strategy-average reward-prediction error $\bar{\delta}^i_t(s,a)$. See Barfuss et al. (2019) for a detailed comparison between the CRLD of SARSA and actor-critic learning.

By trial-and-error, we set the choice intensity of SARSA learning to 60 log-probils per util.

6.1 Compute data

First, we compute the data for the hysteresis curve.

```
# Set up the ecological public goods environment
env = EPG(N=2, f=1.2, c=5, m=-5, qc=0.2, qr=0.01, degraded_choice=False)

# Compile the list of discount factors
dcfs = list(np.arange(0.6, 0.9, 0.005))
# Hysteresis curve parameters first increase and then decrease again
```

```
hystcurve = dcfs + dcfs[::-1]

coops = []  # for storing the cooperation probabilities
for i, dcf in enumerate(hystcurve):
    # Adjust multi-agent environment interface with discount factor
    MAEi = stratS(env=env, discount_factors=dcf, use_prefactor=True,
                  learning_rates=0.01, choice_intensities=60)
    if i==0: # Choose random intial policy
        X = MAEi.random_softmax_strategy()

    # Compute trajectory
    trj, fpr = MAEi.trajectory(X, Tmax=2500, tolerance=10e-12)
    print('\r ', dcf, fpr, end=' ')
    X = trj[-1] # select last strategy
    coops.append(X[:, 1, 0]) # append to storage container
```

6.2 Plot curve

Now, we plot the computed data. We use the points' size and color to indicate the time dimensions of the discount factor changes. The time flows from big to small data points and from dark to light ones.

```
# Create the canves
fsf = 0.75  # figure size factor
plt.figure(figsize=(fsf*6, fsf*3))

# Plot background line
plt.plot(hystcurve, np.array(coops).mean(-1),'-',alpha=0.5,color='k',zorder=-1)
# Plot data points with size and color indicating the time dimension
plt.scatter(hystcurve, np.array(coops).mean(-1), alpha=0.9,
            s=np.arange(len(hystcurve))[::-1]+1, c=np.arange(len(hystcurve)))

# Make labels and axis nice
plt.ylabel('Cooperation')
plt.xlabel('Discount Factor')
plt.gca().spines.right.set_visible(False)
plt.gca().spines.top.set_visible(False)

# Legend
ax = plt.gcf().add_axes([0.85, 0.22, 0.12, 0.6])
# ax = plt.gcf().add_axes([0.135, 0.38, 0.12, 0.6])
ax.scatter(np.ones_like(hystcurve)[::4], np.arange(len(hystcurve))[::4], alpha=0.9,
           s=0.75*np.arange(len(hystcurve))[::-1][::4]+1,
 ↪  c=np.arange(len(hystcurve))[::4])
# ax.annotate('Time', xy=(0.5, 1.07), xycoords='axes fraction', va='center',
 ↪  ha='center', fontsize=9)
ax.annotate('Start', xy=(1.6, 0), xycoords='data', va='center', ha='left',
 ↪  fontsize=8)
ax.annotate('End', xy=(1.6, len(hystcurve)-5), xycoords='data', va='center',
 ↪  ha='left', fontsize=8)
```

```
ax.set_ylim(-10,); ax.set_xlim(0,4)
ax.set_yticks([]); ax.set_xticks([])
for spine in ax.spines.values(): spine.set_edgecolor('grey')

# Save plot
plt.subplots_adjust(left=0.125, right=0.98, top=0.98, bottom=0.2)
plt.savefig("_figs/fig_03Hysteresis.png", dpi=150)
```

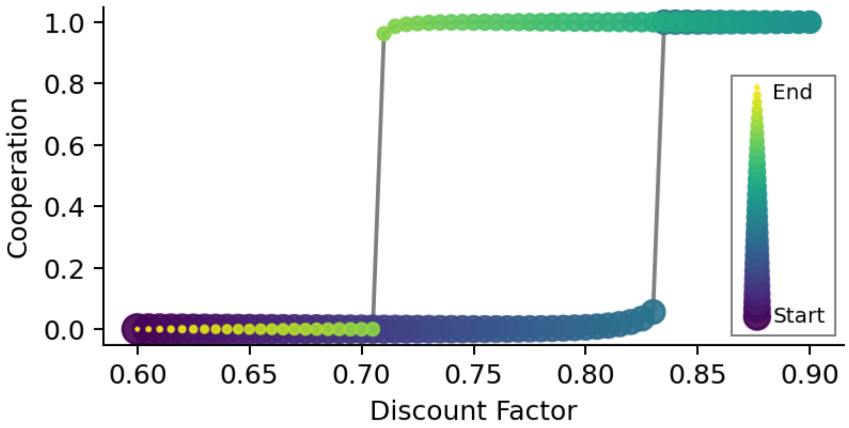

Figure 6.1: Hysteresis curve

As one can see, when the discount factor starts to increase, the learners remain close to defection up to the critical point of about 0.83 when they suddenly switch to complete cooperation. However, when the discount factor starts to decrease again, they remain at almost full cooperation until a much smaller value of approx. 0.71. Only then do the agents suddenly become complete defectors again.

7 Dynamic regimes

This section illustrates that different dynamic regimes with complex transient dynamics can emerge in CRLD. Dynamic regimes are, for example, the convergence to fixed points, continued oscillations, such as limit cycles or periodic orbits, and unpredictable, chaotic dynamics. Thus, they hold the potential for rich, dynamic phenomena beyond the convergence to static equilibria.

First, we import everything we need:

```
import numpy as np
from pyDOE import lhs
import matplotlib.pyplot as plt
from matplotlib.gridspec import GridSpec
from matplotlib.lines import Line2D

from pyCRLD.Environments.SocialDilemma import SocialDilemma as PD
from pyCRLD.Environments.HistoryEmbedding import HistoryEmbedded as he
from pyCRLD.Agents.StrategyActorCritic import stratAC
from pyCRLD.Utils import FlowPlot as fp
```

7.1 Memory-one prisoners' dilemma environment

By trial and error, we saw that the ecological public goods environment is not prone to exhibit dynamic regimes other than convergence to fixed points. Thus, we here use the memory-one Prisoner's Dilemma. We start creating the memory-one Prisoner's Dilemma by initializing a standard normal-form Prisoner's Dilemma.

```
pd = PD(R=1.0, T=1.25, S=-0.25, P=0)
```

The rewards of agent 1 and agent 2, respectively, are:

```
pd.R[0, 0, :, :, 0]
```

```
array([[ 1.  , -0.25],
       [ 1.25,  0.  ]])
```

and

```
pd.R[1, 0, :, :, 0]
```

```
array([[ 1.  ,  1.25],
       [-0.25,  0.  ]])
```

with the first action *cooperation*, and the second *defection*. The environment state set consists only of a void dummy state,

```
pd.Sset
```

```
['.']
```

To transform the normal-form Prisoner's Dilemma (PD) into a memory-one PD, we can use the history-embedding class `he`.

::: {.cell 0='h' 1='i' 2='d' 3='e' execution_count=10}

```
from nbdev.showdoc import *
```

:::

```
show_doc(he)
```

---

source

7.1.1 HistoryEmbedded

```
HistoryEmbedded (env, h)
```

Abstract Environment wrapper to embed a given environment into a larger history space

`h` must be an iterable of length 1+N (where N=Nr. of Agents) The first element of `history` specifies the length of the state-history. Subsequent elements specify the length of the respective action-history

	Details
env	An environment
h	History specification

Thus,

```
# Inititalize the memory-1 Prisoner's Dilemma:
env = he(pd, (1,1,1))
```

which automatically results in the following four environmental states,

```
env.Sset
```

```
['c,c,.|', 'c,d,.|', 'd,c,.|', 'd,d,.|']
```

For example, the first state, 'c,c,.|, indicates that both agents chose cooperation c in the previous round. The state, 'c,d,.|, means that the first agents chose cooperation c and the second defection d, and so on.

With the memory-one PD environment ready, we can finally create the multi-agent environment interface

```
MAEi = stratAC(env=env, learning_rates=0.1, discount_factors=0.99)
```

7.2 Compute data

The initial strategy is vital to showcase different dynamic regimes in the memory-one PD environment. By trial and error (see Section 7.4), we obtained an interesting initial strategy which we hardcoded below. We compare this initial strategy to two other strategies. One is close by, the other further apart.

```
# Initial strategies
# ------------------
# Initial strategy 1
X1 = np.array([[[0.60862106, 0.39137894],
                [0.65139908, 0.34860092],
                [0.72655916, 0.27344087],
                [0.52245504, 0.47754502]],
               [[0.26495466, 0.73504543],
                [0.88308924, 0.1169107 ],
                [0.37133005, 0.62866992],
                [0.53166837, 0.46833161]]])
# Initial strategy 2
X2 = np.array([[[0.60, 0.4],
                [0.6, 0.4],
                [0.7, 0.3],
                [0.5, 0.5]],
               [[0.3, 0.7],
                [0.8, 0.2 ],
                [0.3, 0.7],
                [0.5, 0.5]]])
# Initial strategy 3
Pi = np.array([0.98, 0.05, 0.85, 0.99])
Pj = np.array([0.2, 0.8, 0.05, 0.95])
xi = np.array([Pi, 1-Pi]).T
xj = np.array([Pj, 1-Pj]).T
X3 = np.array([xi, xj])
# Initial strategies
Xs = [X1, X2, X3]

# Trajectories
# ------------
xtrajs = []  # storing strategy trajectories
fprs = []    # and whether a fixed point is reached
for i, X in enumerate(Xs):
    xtraj, fpr = MAEi.trajectory(X, Tmax=2500, tolerance=10**-5)
```

```
    xtrajs.append(xtraj)
    fprs.append(fpr)

# Compute reward trajectories
rtrajs = [np.array([MAEi.Ri(x) for x in xtraj]) for xtraj in xtrajs]
```

7.3 Plot data

We plot the computed data with the strategy trajectories in phase space and reward trajectories over time.

```
# Create canvas
fsf = 0.7  # figure size factor
fig = plt.figure(figsize=(fsf*4.5, fsf*6))
gs = GridSpec(2, 1, height_ratios=[1, 4],
              hspace=0.35, left=0.18, right=0.98, top=0.92, bottom=0.12)
ax1 = fig.add_subplot(gs[0])
ax2 = fig.add_subplot(gs[1])

# Strategy flow plot
# ------------------
x = ([0], [0], [0])  # which (agent, observation, action) to plot on x axis
y = ([1], [0], [0])  # which (agent, observation, action) to plot on y axis
eps=10e-3; action_probability_points = np.linspace(0+eps, 1.0-eps, 9)
fp.plot_strategy_flow(MAEi, x, y, action_probability_points, NrRandom=64,
                      cmap='Greys', sf=0.20, axes=[ax2])

# Trajectories
# ------------
# in phase space
fp.plot_trajectories(xtrajs, x=x, y=y, fprs=fprs, axes=[ax2], alphas=[0.5],
                     cols=['red','orange','blue'], lws=[2], lss=['-'])
# and over time
ax1.plot(rtrajs[0][:, 0], c='red', ls='--')
ax1.plot(rtrajs[0][:, 1], c='red', ls=':')
ax1.plot(rtrajs[1][:, 0], c='orange', ls='--')
ax1.plot(rtrajs[1][:, 1], c='orange', ls=':')
ax1.plot(rtrajs[2][:, 0], c='blue', ls='--')
ax1.plot(rtrajs[2][:, 1], c='blue', ls=':')

# Decorations
# -----------
# Make labels nice
ax1.set_ylim(-0.25, 1.25)
ax1.set_ylabel('Reward')
ax1.set_xlabel('Time steps')
ax2.set_ylabel(f"$X^2(s=CC,a=C)$")
ax2.set_xlabel(f"$X^1(s=CC,a=C)$")

# Create legend
```

```
custom_lines = [Line2D([0], [0], color='gray', ls='--', lw=1),
                Line2D([0], [0], color='gray', ls=':', lw=1)]
ax1.legend(custom_lines, ['Agent 1', 'Agent 2'], ncol=2, bbox_to_anchor=(1,1),
           loc='lower right')

# Save plot
plt.savefig('_figs/fig_04DynamicRegimes.png', dpi=150)
```

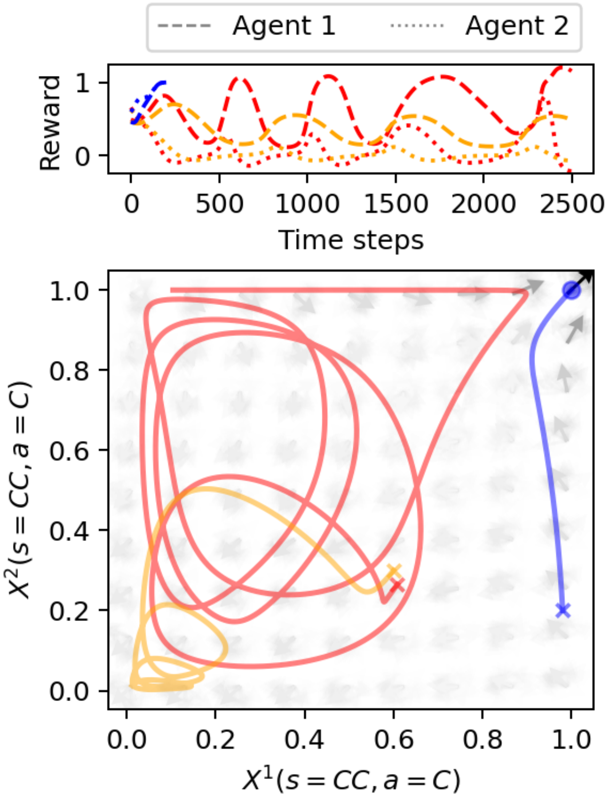

Figure 7.1: Different dynamic regimes in the phase space of the memory-1 Prisoners' Dilemma

7.4 Find initial strategy

Last, we show how to find an initial strategy that leads to a dynamic regime other than the convergence to a fixed point.

```
# If necessary, create  multi-agent environment interface from scratch
env = he(PD(R=1.0, T=1.25, S=-0.25, P=0), (1,1,1))
MAEi = stratAC(env=env, learning_rates=0.1, discount_factors=0.99)

# Compute trajectories for some random initial strategies
# and see whether they did not reach a fixed point.
print("No fixed point reached for trajectories:")
xtrajs = []
Tmax = 2500 # Convergence-time threshold
for i in range(500):
    X = MAEi.random_softmax_strategy()
```

```
    xtraj, fpr = MAEi.trajectory(X, Tmax=Tmax, tolerance=10**-5)
    xtrajs.append(xtraj)

    if not fpr:
        print(i, end=' ')
```

```
No fixed point reached for trajectories:
142 238 487
```

We check that the center of the distribution of convergence times is well below the convergence-time threshold.

```
plt.figure(figsize=(3,2))
hist = plt.hist([len(xt) for xt in xtrajs], bins=25)
plt.plot([Tmax, Tmax], [0, 1.1*max(hist[0])], c='red')
plt.ylim(0, 1.1*max(hist[0]))
plt.xlabel("Convergence timesteps")
plt.ylabel("Count");
```

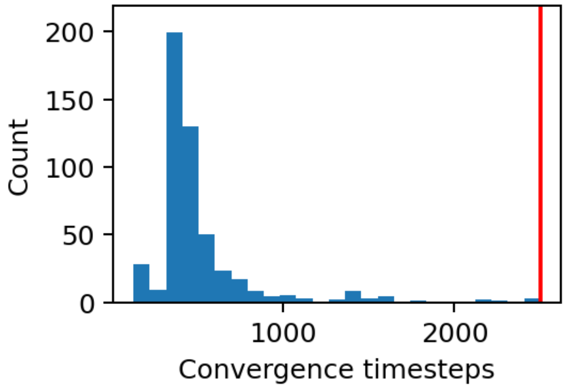

Figure 7.2: Histogram of convergence times.

Last, we examine the non-convergent trajectories in the strategy phase space of all environmental states of the memory-one Prisoner's Dilemma.

```
xt, fpr = MAEi.trajectory(xtrajs[238][0], Tmax=2500, tolerance=10**-5)

x = ([0], [0,1,2,3], [0])  # which (agent, observation, action) to plot on x ax
y = ([1], [0,1,2,3], [0])  # which (agent, observation, action) to plot on y ax
eps=10e-3; action_probability_points = np.linspace(0+eps, 1.0-eps, 9)
ax = fp.plot_strategy_flow(MAEi, x, y, action_probability_points, NrRandom=32,
                           conds=MAEi.env.Sset)
fp.plot_trajectories([xt], x=x, y=y,
                     cols=['red'], lws=[2], lss=['-'], alphas=[0.5], axes=ax);
```

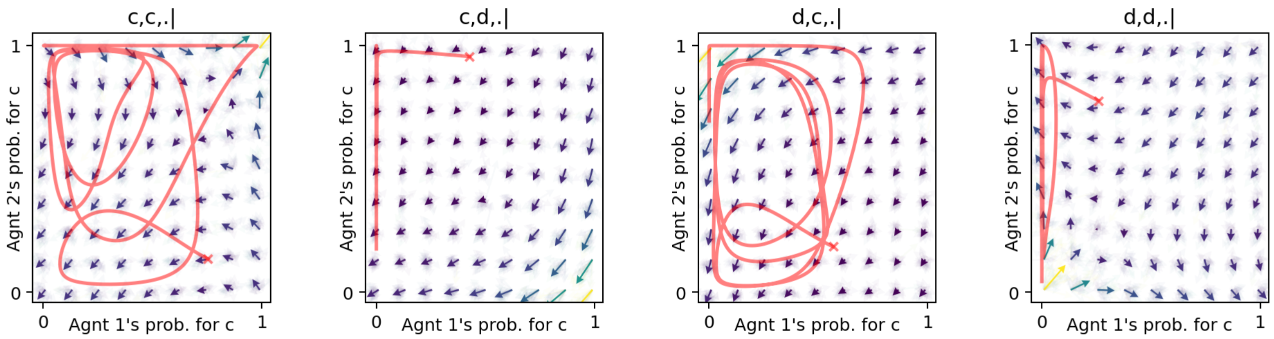

c,c,.|
c,d,.|
d,c,.|
d,d,.|
Agnt 1's prob. for c
Agnt 2's prob. for c
0
1

8 Simulation Scripts

Here we introduce scripts which support the simulation of CRLD.

```
#| hide
from nbdev.showdoc import *
```

```
#| default_exp SimulationScripts
```

```
#| export
import numpy as np
import matplotlib.pyplot as plt
from skopt.sampler import Lhs
from skopt.space import Space
import hashlib
```

8.1 Create initial strategies

```
#| exports
def initial_strategies(MAEi,  # Multi-agent environment interface
                       number:int,  # Number of strategies to create
                       iterations:int=1000  # Latin hyper cube sampling parameter
                       )->np.ndarray:  # Array of initial strategies
    """
    Create a set of inital strategies using latin hyper cube sampling
    """
    assert MAEi.M == 2, 'Sampling for M>2 not straightforward'
    # https://www.egr.msu.edu/~kdeb/papers/c2018010.pdf
    # https://www.cs.cmu.edu/~nasmith/papers/smith+tromble.tr04.pdf

    eps = 10**(-6)
    space = Space(MAEi.N * MAEi.Q * (MAEi.M-1)*[(0.0+eps, 1.0-eps)])

    # generate latin hyper cubes
    lhs = Lhs(criterion="maximin", iterations=iterations)
    x = lhs.generate(space.dimensions, number, random_state=42)
    x = np.array(x).reshape(number, MAEi.N, MAEi.Q, MAEi.M-1)

    # complete and normalize
    inits = np.zeros((number, MAEi.N, MAEi.Q, MAEi.M))
    inits[..., 0] = x[...,0]
    inits[..., 1] = 1 - x[...,0]
```

```
    return inits
```

For example,

```
class mae: N=3; Q=4; M=2 # dummy MAEi for demonstration only
Xs = initial_strategies(mae, 7)
Xs.shape
```

```
(7, 3, 4, 2)
```

8.2 Compute trajectories

```
#| exports
def compute_trajectories(MAEi,  # Multi-agent environment interface
                         inits,  # Iterable of inital conditions
                         Tmax=1000,  # Number of maximum iteration steps for each
↪ run
                         tol=10e-5  # Tolerance to classify a trajectory as
↪ converged
                         )->tuple:  # (iterables of trajectories,
↪ fixed-point-reacheds)
    """
    Compute learning trajectories from a set of inital strategies.
    """
    trjs = []; fprs = []
    leni = len(inits)

    for xi, x0 in enumerate(inits):
        print("\r ", np.round(xi/leni, 4), end='')
        x = x0.copy()

        trj, fpr = MAEi.trajectory(x, Tmax=Tmax, tolerance=tol)

        trjs.append(trj)
        fprs.append(fpr)

    print()
    print('Computed', leni, 'trajectories')

    return np.array(trjs, dtype=object), np.array(fprs)
```

After computing the trajectories, we can check whether or not all converged and look at the histograms of their lengths:

```
#| exports
def check_run(trjs,  # Iterable of learning trajectories
              fprs=None):  # Iterable of bools whether a fixed point was reached
    """
    Perform some checks for an iterable of learning trajectories
    """
    if fprs is not None:
        print('Unique fixed points reached:', np.unique(fprs))
    plt.hist([len(traj) for traj in trjs], bins=20);
    plt.title('Histrogram of trajectories lengths')
```

8.3 Saving & reloading

To not recompute everything from scratch, we save runs to disk and retrieve them more efficiently and faster when needed.

```
#| exports
def _transform_tensor_into_hash(tens):
    """Transform `tens` into a string for filename saving"""
    r = int(hashlib.sha512(str(tens).encode('utf-8')).hexdigest()[:16], 16)
    return r
```

```
#| exports
def obtain_trajectories(MAEi,  # Multi-agent environment interface
                        inits,  # Iterable of inital conditions
                        Tmax=1000,  # Number of maximum iteration steps for each run
                        tol=10e-5,  # Tolerance to classify a trajectory as
↪   converged
                        ddir='data',  # Path to data directory to store the results
                        verbose=1  # Verbosity level
                        )->tuple:  # (iterables of trajectories,
↪   fixed-point-reacheds)
    """
    Obtain learning trajectories from a set of inital strategies.
    Check wether you can load them from disk. If yes, do so. If not, compute.
    """
    fn = ddir + '/' + MAEi.id() + '_' + str(_transform_tensor_into_hash(inits))
    fn += ".npz"

    try:
        dat = np.load(fn, allow_pickle=True)
        ddic = dict(zip((k for k in dat), (dat[k] for k in dat)))
        print("Loading ", fn) if verbose else None

    except:
        print("Computing ", fn) if verbose else None
        trjs, fprs = compute_trajectories(MAEi, inits, Tmax=Tmax, tol=tol)
        check_run(trjs, fprs)
        # rtrajs = obtain_rewards(AEi, trajs)
```

```
        ddic = dict(trjs=trjs, fprs=fprs)
        np.savez_compressed(fn, **ddic)
        dat = np.load(fn, allow_pickle=True)
        ddic = dict(zip((k for k in dat), (dat[k] for k in dat)))

    return ddic['trjs'], ddic['fprs']
```

8.4 Final rewards

```
#| exports
def final_rewards(MAEi, # Multi-agent environment interface
                  trjs  # Iterable of learning trajectories
                  )->np.ndarray:  # Array of final rewards
    """
    Compute final rewards from a set of learning trajectories.
    """
    rews = []
    for trj in trjs:
        x = trj[-1].astype(float)
        rs = np.einsum(MAEi.Ps(x), [0], MAEi.Ris(x), [1,0], [1])
        # rs = MAEi.Ri(x)
        rews.append(rs)

    return np.array(rews)
```

```
#| hide
import nbdev
nbdev.export.nb_export("08_SimulationScripts.ipynb", "_code")
```

9 References

Arthur, W. B. (1994). Inductive reasoning and bounded rationality. *The American Economic Review*, *84*(2), 406–411.

Arthur, W. B. (2014). *Complexity and the Economy*. Oxford University Press.

Axelrod, R. (1984). *The Evolution Of Cooperation*. Basic Books.

Axelrod, R., & Hamilton, W. D. (1981). The Evolution of Cooperation. *Science*, *211*(4489), 1390–1396. https://doi.org/10.1126/science.7466396

Barfuss, W. (2020). Reinforcement Learning Dynamics in the Infinite Memory Limit. *Proceedings of the 19th International Conference on Autonomous Agents and MultiAgent Systems*, 1768–1770.

Barfuss, W. (2022). Dynamical systems as a level of cognitive analysis of multi-agent learning. *Neural Computing and Applications*, *34*(3), 1653–1671. https://doi.org/10.1007/s00521-021-06117-0

Barfuss, W., Donges, J. F., & Kurths, J. (2019). Deterministic limit of temporal difference reinforcement learning for stochastic games. *Physical Review E*, *99*(4), 043305. https://doi.org/10.1103/PhysRevE.99.043305

Barfuss, W., Donges, J. F., Vasconcelos, V. V., Kurths, J., & Levin, S. A. (2020). Caring for the future can turn tragedy into comedy for long-term collective action under risk of collapse. *Proceedings of the National Academy of Sciences*, *117*(23), 12915–12922. https://doi.org/10.1073/pnas.1916545117

Berner, C., Brockman, G., Chan, B., Cheung, V., Dębiak, P., Dennison, C., Farhi, D., Fischer, Q., Hashme, S., Hesse, C., Józefowicz, R., Gray, S., Olsson, C., Pachocki, J., Petrov, M., Pinto, H. P. d. O., Raiman, J., Salimans, T., Schlatter, J., … Zhang, S. (2019). *Dota 2 with Large Scale Deep Reinforcement Learning* (arXiv:1912.06680). arXiv. https://doi.org/10.48550/arXiv.1912.06680

Bialek, W., Cavagna, A., Giardina, I., Mora, T., Silvestri, E., Viale, M., & Walczak, A. M. (2012). Statistical mechanics for natural flocks of birds. *Proceedings of the National Academy of Sciences*, *109*(13), 4786–4791. https://doi.org/10.1073/pnas.1118633109

Botvinick, M., Wang, J. X., Dabney, W., Miller, K. J., & Kurth-Nelson, Z. (2020). Deep Reinforcement Learning and Its Neuroscientific Implications. *Neuron*, *107*(4), 603–616. https://doi.org/10.1016/j.neuron.2020.06.014

Brush, E. R., Krakauer, D. C., & Flack, J. C. (2018). Conflicts of interest improve collective computation of adaptive social structures. *Science Advances*, *4*(1), e1603311. https://doi.org/10.1126/sciadv.1603311

Buckley, C. L., Kim, C. S., McGregor, S., & Seth, A. K. (2017). The free energy principle for action and perception: A mathematical review. *Journal of Mathematical Psychology*, *81*, 55–79. https://doi.org/10.1016/j.jmp.2017.09.004

Buhl, J., Sumpter, D. J. T., Couzin, I. D., Hale, J. J., Despland, E., Miller, E. R., & Simpson, S. J. (2006). From Disorder to Order in Marching Locusts. *Science*, *312*(5778), 1402–1406. https://doi.org/10.1126/science.1125142

Bush, R. R., & Mosteller, F. (1951). A mathematical model for simple learning. *Psychological Review*, *58*, 313–323. https://doi.org/10.1037/h0054388

Busoniu, L., Babuska, R., & De Schutter, B. (2008). A comprehensive survey of multiagent reinforcement learning. *IEEE Transactions on Systems, Man, and Cybernetics, Part C (Applications and Reviews)*, *38*(2), 156–172.

Camerer, C. F. (2011). *Behavioral game theory: Experiments in strategic interaction*. Princeton university press.

Carroll, M., Shah, R., Ho, M. K., Griffiths, T., Seshia, S., Abbeel, P., & Dragan, A. (2019). On the utility of learning about humans for human-ai coordination. *Advances in Neural Information*

Processing Systems, *32*.

Christoffersen, P. J., Haupt, A. A., & Hadfield-Menell, D. (2022). Get it in writing: Formal contracts mitigate social dilemmas in multi-agent RL. *arXiv Preprint arXiv:2208.10469*.

Cohen, J. E. (1998). Cooperation and self-interest: Pareto-inefficiency of Nash equilibria in finite random games. *Proceedings of the National Academy of Sciences*, *95*(17), 9724–9731. https://doi.org/10.1073/pnas.95.17.9724

Cross, J. G. (1973). A Stochastic Learning Model of Economic Behavior*. *The Quarterly Journal of Economics*, *87*(2), 239–266. https://doi.org/10.2307/1882186

Dafoe, A., Bachrach, Y., Hadfield, G., Horvitz, E., Larson, K., & Graepel, T. (2021). Cooperative AI: Machines must learn to find common ground. *Nature*, *593*(7857), 33–36. https://doi.org/10.1038/d41586-021-01170-0

Daniels, B. C., Ellison, C. J., Krakauer, D. C., & Flack, J. C. (2016). Quantifying collectivity. *Current Opinion in Neurobiology*, *37*, 106–113. https://doi.org/10.1016/j.conb.2016.01.012

Daniels, B. C., Krakauer, D. C., & Flack, J. C. (2017). Control of finite critical behaviour in a small-scale social system. *Nature Communications*, *8*(1), 14301. https://doi.org/10.1038/ncomms14301

Daniels, B. C., Laubichler, M. D., & Flack, J. C. (2021). Introduction to the special issue: Quantifying collectivity. *Theory in Biosciences*, *140*(4), 321–323. https://doi.org/10.1007/s12064-021-00358-2

Darriba, Á., & Waszak, F. (2018). Predictions through evidence accumulation over time. *Scientific Reports*, *8*(1), 494. https://doi.org/10.1038/s41598-017-18802-z

Dawes, R. M. (1980). Social Dilemmas. *Annual Review of Psychology*, *31*(1), 169–193. https://doi.org/10.1146/annurev.ps.31.020180.001125

Dayan, P., & Niv, Y. (2008). Reinforcement learning: The Good, The Bad and The Ugly. *Current Opinion in Neurobiology*, *18*(2), 185–196. https://doi.org/10.1016/j.conb.2008.08.003

De Marzo, G., Gabrielli, A., Zaccaria, A., & Pietronero, L. (2022). Quantifying the unexpected: A scientific approach to Black Swans. *Physical Review Research*, *4*(3), 033079. https://doi.org/10.1103/PhysRevResearch.4.033079

DeDeo, S., Krakauer, D. C., & Flack, J. C. (2010). Inductive game theory and the dynamics of animal conflict. *PLoS Computational Biology*, *6*(5), e1000782.

Epstein, J. M., & Axtell, R. L. (1996). *Growing Artificial Societies: Social Science From the Bottom Up* (First Edition). Brookings Institution Press.

Erev, I., & Roth, A. E. (1998). Predicting How People Play Games: Reinforcement Learning in Experimental Games with Unique, Mixed Strategy Equilibria. *The American Economic Review*, *88*(4), 848–881.

(FAIR)†, M. F. A. R. D. T., Bakhtin, A., Brown, N., Dinan, E., Farina, G., Flaherty, C., Fried, D., Goff, A., Gray, J., Hu, H., et al. (2022). Human-level play in the game of diplomacy by combining language models with strategic reasoning. *Science*, *378*(6624), 1067–1074.

Fehr, E., & Gächter, S. (2000). Cooperation and Punishment in Public Goods Experiments. *American Economic Review*, *90*(4), 980–994. https://doi.org/10.1257/aer.90.4.980

Flack, J. C. (2017). Coarse-graining as a downward causation mechanism. *Philosophical Transactions of the Royal Society A: Mathematical, Physical and Engineering Sciences*, *375*(2109), 20160338. https://doi.org/10.1098/rsta.2016.0338

Foerster, J., Assael, I. A., de Freitas, N., & Whiteson, S. (2016). Learning to Communicate with Deep Multi-Agent Reinforcement Learning. *Advances in Neural Information Processing Systems*, *29*.

Franci, A., Golubitsky, M., Stewart, I., Bizyaeva, A., & Leonard, N. E. (2022). Breaking indecision in multi-agent, multi-option dynamics. *arXiv Preprint arXiv:2206.14893*.

Friston, K. (2018). Does predictive coding have a future? *Nature Neuroscience*, *21*(8), 1019–1021. https://doi.org/10.1038/s41593-018-0200-7

Fudenberg, D., & Levine, D. K. (1998). *The Theory of Learning in Games* (K. Binmore, Ed.). MIT Press.

Grupen, N., Jaques, N., Kim, B., & Omidshafiei, S. (2022). Concept-based understanding of emergent multi-agent behavior. *Deep Reinforcement Learning Workshop NeurIPS 2022*. https://openreview.net/forum?id=zt5JpGQ8WhH

Gunawardena, J. (2022). Learning Outside the Brain: Integrating Cognitive Science and Systems Biology. *Proceedings of the IEEE*, 1–23. https://doi.org/10.1109/JPROC.2022.3162791

Hauert, C. (2002). Effects of space in 2 × 2 games. *International Journal of Bifurcation and Chaos*, *12*(07), 1531–1548. https://doi.org/10.1142/S0218127402005273

Hauert, C., & Doebeli, M. (2004). Spatial structure often inhibits the evolution of cooperation in the snowdrift game. *Nature*, *428*(6983), 643–646. https://doi.org/10.1038/nature02360

Hauert, C., Michor, F., Nowak, M. A., & Doebeli, M. (2006). Synergy and discounting of cooperation in social dilemmas. *Journal of Theoretical Biology*, *239*(2), 195–202. https://doi.org/10.1016/j.jtbi.2005.08.040

Hauser, O. P., Hilbe, C., Chatterjee, K., & Nowak, M. A. (2019). Social dilemmas among unequals. *Nature*, *572*(7770), 524–527.

Heins, C., Millidge, B., Costa, L. da, Mann, R., Friston, K., & Couzin, I. (2023). *Collective behavior from surprise minimization.* arXiv. http://arxiv.org/abs/2307.14804

Hernandez-Leal, P., Kartal, B., & Taylor, M. E. (2019). A survey and critique of multiagent deep reinforcement learning. *Autonomous Agents and Multi-Agent Systems*, *33*(6), 750–797. https://doi.org/10.1007/s10458-019-09421-1

Hilbe, C., Chatterjee, K., & Nowak, M. A. (2018). Partners and rivals in direct reciprocity. *Nature Human Behaviour*. https://doi.org/10.1038/s41562-018-0320-9

Hilbe, C., Šimsa, Š., Chatterjee, K., & Nowak, M. A. (2018). Evolution of cooperation in stochastic games. *Nature*, *559*(7713), 246–249. https://doi.org/10.1038/s41586-018-0277-x

Hofbauer, J., & Sigmund, K. (1998). *Evolutionary Games and Population Dynamics* (First). Cambridge University Press. https://doi.org/10.1017/CBO9781139173179

Hofbauer, J., & Sigmund, K. (2003). Evolutionary game dynamics. *Bulletin of the American Mathematical Society*, *40*(4), 479–519. https://doi.org/10.1090/S0273-0979-03-00988-1

Holland, J. H., & Miller, J. H. (1991). Artificial Adaptive Agents in Economic Theory. *The American Economic Review*, *81*(2), 365–370.

Hughes, E., Anthony, T. W., Eccles, T., Leibo, J. Z., Balduzzi, D., & Bachrach, Y. (2020). Learning to Resolve Alliance Dilemmas in Many-Player Zero-Sum Games. *New Zealand*, 10.

Jaynes, E. T., & Bretthorst, G. L. (2003). *Probability theory: The logic of science.* Cambridge University Press. http://www5.unitn.it/Biblioteca/it/Web/LibriElettroniciDettaglio/50847

Jhawar, J., Morris, R. G., Amith-Kumar, U. R., Danny Raj, M., Rogers, T., Rajendran, H., & Guttal, V. (2020). Noise-induced schooling of fish. *Nature Physics*, *16*(4), 488–493. https://doi.org/10.1038/s41567-020-0787-y

Kempes, C. P., Wolpert, D., Cohen, Z., & Pérez-Mercader, J. (2017). The thermodynamic efficiency of computations made in cells across the range of life. *Philosophical Transactions of the Royal Society A: Mathematical, Physical and Engineering Sciences*, *375*(2109), 20160343. https://doi.org/10.1098/rsta.2016.0343

Kleshnina, M., Hilbe, C., Šimsa, Š., Chatterjee, K., & Nowak, M. A. (2023). The effect of environmental information on evolution of cooperation in stochastic games. *Nature Communications*, *14*(1), 4153. https://doi.org/10.1038/s41467-023-39625-9

Krakauer, D. C., Flack, J. C., Dedeo, S., Farmer, D., & Rockmore, D. (2010). Intelligent Data Analysis of Intelligent Systems. In P. R. Cohen, N. M. Adams, & M. R. Berthold (Eds.), *Advances in Intelligent Data Analysis IX* (pp. 8–17). Springer. https://doi.org/10.1007/978-3-642-13062-5_3

Krakauer, D., Bertschinger, N., Olbrich, E., Flack, J. C., & Ay, N. (2020). The information theory of individuality. *Theory in Biosciences*, *139*(2), 209–223. https://doi.org/10.1007/s12064-020-00313-7

Leibo, J. Z., Dueñez-Guzman, E. A., Vezhnevets, A., Agapiou, J. P., Sunehag, P., Koster, R., Matyas, J., Beattie, C., Mordatch, I., & Graepel, T. (2021). Scalable evaluation of multi-agent reinforcement learning with melting pot. *International Conference on Machine Learning*, 6187–6199.

Leibo, J. Z., Zambaldi, V., Lanctot, M., Marecki, J., & Graepel, T. (2017). Multi-agent Reinforcement Learning in Sequential Social Dilemmas. *Proceedings of the 16th Conference on Autonomous Agents and MultiAgent Systems*, 464–473.

Levin, S. (2002). Complex adaptive systems: Exploring the known, the unknown and the unknowable. *Bulletin of the American Mathematical Society*, *40*(1), 3–19. https://doi.org/10.1090/S0273-0979-02-00965-5

Littman, M. L. (1994). Markov games as a framework for multi-agent reinforcement learning. In W. W. Cohen & H. Hirsh (Eds.), *Machine Learning Proceedings 1994* (pp. 157–163). Morgan Kaufmann. https://doi.org/10.1016/B978-1-55860-335-6.50027-1

Lovering, C., Forde, J., Konidaris, G., Pavlick, E., & Littman, M. (2022). Evaluation beyond task performance: Analyzing concepts in AlphaZero in hex. In S. Koyejo, S. Mohamed, A. Agarwal, D. Belgrave, K. Cho, & A. Oh (Eds.), *Advances in neural information processing systems* (Vol. 35, pp. 25992–26006). Curran Associates, Inc. https://proceedings.neurips.cc/paper_files/paper/2022/file/a705747417d32ebf1916169e1a442274-Paper-Conference.pdf

Lupu, A., & Precup, D. (2020). Gifting in multi-agent reinforcement learning. *Proceedings of the 19th International Conference on Autonomous Agents and Multiagent Systems*, 789–797.

Marden, J. R., & Shamma, J. S. (2018). Game theory and control. *Annual Review of Control, Robotics, and Autonomous Systems*, *1*, 105–134.

McAvoy, A., Mori, Y., & Plotkin, J. B. (2022). Selfish optimization and collective learning in populations. *Physica D: Nonlinear Phenomena*, *439*, 133426. https://doi.org/10.1016/j.physd.2022.133426

McGrath, T., Kapishnikov, A., Tomašev, N., Pearce, A., Wattenberg, M., Hassabis, D., Kim, B., Paquet, U., & Kramnik, V. (2022). Acquisition of chess knowledge in AlphaZero. *Proceedings of the National Academy of Sciences*, *119*(47), e2206625119.

McNamara, J. M. (2013). Towards a richer evolutionary game theory. *Journal of The Royal Society Interface*, *10*(88), 20130544. https://doi.org/10.1098/rsif.2013.0544

McNamara, J. M., Houston, A. I., & Leimar, O. (2021). Learning, exploitation and bias in games. *PLOS ONE*, *16*(2), e0246588. https://doi.org/10.1371/journal.pone.0246588

Mora, T., & Bialek, W. (2011). Are Biological Systems Poised at Criticality? *Journal of Statistical Physics*, *144*(2), 268–302. https://doi.org/10.1007/s10955-011-0229-4

Newman, M. E. J. (2003). The Structure and Function of Complex Networks. *SIAM Review*, *45*(2), 167–256. https://doi.org/10.1137/S003614450342480

Nowak, M. A. (2006). *Evolutionary dynamics: Exploring the equations of life*. Harvard university press.

Ostrom, E., Walker, J., & Gardner, R. (1992). Covenants with and without a Sword: Self-Governance Is Possible. *American Political Science Review*, *86*(2), 404–417. https://doi.org/10.2307/1964229

Park, S., Bizyaeva, A., Kawakatsu, M., Franci, A., & Leonard, N. E. (2021). Tuning cooperative behavior in games with nonlinear opinion dynamics. *IEEE Control Systems Letters*, *6*, 2030–2035.

Poundstone, W. (2011). *Prisoner's Dilemma*. Knopf Doubleday Publishing Group.

Press, W. H., & Dyson, F. J. (2012). Iterated prisoner's dilemma contains strategies that dominate any evolutionary opponent. *Proceedings of the National Academy of Sciences*, *109*(26), 10409–10413. https://doi.org/10.1073/pnas.1206569109

Ramos-Fernandez, G., Smith Aguilar, S. E., Krakauer, D. C., & Flack, J. C. (2020). Collective Computation in Animal Fission-Fusion Dynamics. *Frontiers in Robotics and AI*, *7*. https://www.frontiersin.org/article/10.3389/frobt.2020.00090

Rao, R. P. N., & Ballard, D. H. (1999). Predictive coding in the visual cortex: A functional interpretation of some extra-classical receptive-field effects. *Nature Neuroscience*, *2*(1), 79–87. https://doi.org/10.1038/4580

Rosas, F. E., Mediano, P. A. M., Gastpar, M., & Jensen, H. J. (2019). Quantifying high-order interdependencies via multivariate extensions of the mutual information. *Physical Review E*, *100*(3), 032305. https://doi.org/10.1103/PhysRevE.100.032305

Roth, A. E., & Erev, I. (1995). Learning in extensive-form games: Experimental data and simple dynamic models in the intermediate term. *Games and Economic Behavior*, *8*(1), 164–212. https://doi.org/10.1016/S0899-8256(05)80020-X

Sarfati, R., Hayes, J. C., & Peleg, O. (2021). Self-organization in natural swarms of Photinus carolinus

synchronous fireflies. *Science Advances*, *7*(28), eabg9259. https://doi.org/10.1126/sciadv.abg9259

Schultz, W., Dayan, P., & Montague, P. R. (1997). A Neural Substrate of Prediction and Reward. *Science*, *275*(5306), 1593–1599. https://doi.org/10.1126/science.275.5306.1593

Schultz, W., Stauffer, W. R., & Lak, A. (2017). The phasic dopamine signal maturing: From reward via behavioural activation to formal economic utility. *Current Opinion in Neurobiology*, *43*, 139–148. https://doi.org/10.1016/j.conb.2017.03.013

Shoham, Y., Powers, R., & Grenager, T. (2007). If multi-agent learning is the answer, what is the question? *Artificial Intelligence*, *171*(7), 365–377. https://doi.org/10.1016/j.artint.2006.02.006

Sigmund, K. (2010). The Calculus of Selfishness. In *The Calculus of Selfishness*. Princeton University Press. https://doi.org/10.1515/9781400832255

Silver, D., Huang, A., Maddison, C. J., Guez, A., Sifre, L., van den Driessche, G., Schrittwieser, J., Antonoglou, I., Panneershelvam, V., Lanctot, M., Dieleman, S., Grewe, D., Nham, J., Kalchbrenner, N., Sutskever, I., Lillicrap, T., Leach, M., Kavukcuoglu, K., Graepel, T., & Hassabis, D. (2016). Mastering the game of Go with deep neural networks and tree search. *Nature*, *529*(7587), 484–489. https://doi.org/10.1038/nature16961

Skyrms, B. (2004). *The Stag Hunt and the Evolution of Social Structure*. Cambridge University Press.

Stone, P., Kaminka, G., Kraus, S., & Rosenschein, J. (2010). Ad hoc autonomous agent teams: Collaboration without pre-coordination. *Proceedings of the AAAI Conference on Artificial Intelligence*, *24*, 1504–1509.

Strouse, D., McKee, K. R., Botvinick, M. M., Hughes, E., & Everett, R. (2021). Collaborating with humans without human data. *CoRR*, *abs/2110.08176*. https://arxiv.org/abs/2110.08176

Sugden, R. (2004). *The Economics of Rights, Co-operation and Welfare*. Springer.

Sutton, R. S. (1988). Learning to predict by the methods of temporal differences. *Machine Learning*, *3*(1), 9–44. https://doi.org/10.1007/BF00115009

Sutton, R. S., & Barto, A. G. (2018). *Reinforcement learning: An introduction* (Second edition). The MIT Press.

Team, C. G. I., Bhoopchand, A., Brownfield, B., Collister, A., Lago, A. D., Edwards, A., Everett, R., Frechette, A., Oliveira, Y. G., Hughes, E., Mathewson, K. W., Mendolicchio, P., Pawar, J., Pislar, M., Platonov, A., Senter, E., Singh, S., Zacherl, A., & Zhang, L. M. (2022). *Learning robust real-time cultural transmission without human data*. https://arxiv.org/abs/2203.00715

Tekin, E., Savage, V. M., & Yeh, P. J. (2017). Measuring higher-order drug interactions: A review of recent approaches. *Current Opinion in Systems Biology*, *4*, 16–23. https://doi.org/10.1016/j.coisb.2017.05.015

Tekin, E., Yeh, P. J., & Savage, V. M. (2018). General Form for Interaction Measures and Framework for Deriving Higher-Order Emergent Effects. *Frontiers in Ecology and Evolution*, *6*. https://www.frontiersin.org/articles/10.3389/fevo.2018.00166

Tuyls, K., Perolat, J., Lanctot, M., Hughes, E., Everett, R., Leibo, J. Z., Szepesvári, C., & Graepel, T. (2019). Bounds and dynamics for empirical game theoretic analysis. *Autonomous Agents and Multi-Agent Systems*, *34*(1), 7. https://doi.org/10.1007/s10458-019-09432-y

Vinyals, O., Babuschkin, I., Czarnecki, W. M., Mathieu, M., Dudzik, A., Chung, J., Choi, D. H., Powell, R., Ewalds, T., Georgiev, P., Oh, J., Horgan, D., Kroiss, M., Danihelka, I., Huang, A., Sifre, L., Cai, T., Agapiou, J. P., Jaderberg, M., … Silver, D. (2019). Grandmaster level in StarCraft II using multi-agent reinforcement learning. *Nature*, *575*(7782), 350–354. https://doi.org/10.1038/s41586-019-1724-z

Wang, W. Z., Beliaev, M., Bıyık, E., Lazar, D. A., Pedarsani, R., & Sadigh, D. (2021). Emergent Prosociality in Multi-Agent Games Through Gifting. *Twenty-Ninth International Joint Conference on Artificial Intelligence*, *1*, 434–442. https://doi.org/10.24963/ijcai.2021/61

Wang, X., & Fu, F. (2020). Eco-evolutionary dynamics with environmental feedback: Cooperation in a changing world. *Europhysics Letters*, *132*(1), 10001. https://doi.org/10.1209/0295-5075/132/10001

Wolfram, S. (1994). *Cellular Automata And Complexity: Collected Papers* (1st edition). Westview Press.

Wolpert, D. H. (2006). Information Theory - The Bridge Connecting Bounded Rational Game Theory and Statistical Physics. In D. Braha, A. A. Minai, & Y. Bar-Yam (Eds.), *Complex Engineered Systems: Science Meets Technology* (pp. 262–290). Springer. https://doi.org/10.1007/3-540-32834-3_12

Wolpert, D. H., Harré, M., Olbrich, E., Bertschinger, N., & Jost, J. (2012). Hysteresis effects of changing the parameters of noncooperative games. *Physical Review E*, *85*(3), 036102. https://doi.org/10.1103/PhysRevE.85.036102
